# A high scale SARS-CoV-2 profiling by its whole-genome sequencing using Oxford Nanopore Technology in Kazakhstan

**DOI:** 10.3389/fgene.2022.906318

**Published:** 2022-09-02

**Authors:** Ulykbek Kairov, Amina Amanzhanova, Daniyar Karabayev, Saule Rakhimova, Akbota Aitkulova, Diana Samatkyzy, Ruslan Kalendar, Ulan Kozhamkulov, Askhat Molkenov, Aidana Gabdulkayum, Dos Sarbassov, Ainur Akilzhanova

**Affiliations:** ^1^ Center for Life Sciences, National Laboratory Astana, Nazarbayev University, Nur-Sultan, Kazakhstan; ^2^ School of Sciences and Humanities, Nazarbayev University, Nur-Sultan, Kazakhstan

**Keywords:** Kazakhstan, third-generation sequencing, Oxford Nanopore, SARS-CoV-2 genome sequencing, COVID-19

## Abstract

Severe acute respiratory syndrome (SARS-CoV-2) is responsible for the worldwide pandemic, COVID-19. The original viral whole-genome was sequenced by a high-throughput sequencing approach from the samples obtained from Wuhan, China. Real-time gene sequencing is the main parameter to manage viral outbreaks because it expands our understanding of virus proliferation, spread, and evolution. Whole-genome sequencing is critical for SARS-CoV-2 variant surveillance, the development of new vaccines and boosters, and the representation of epidemiological situations in the country. A significant increase in the number of COVID-19 cases confirmed in August 2021 in Kazakhstan facilitated a need to establish an effective and proficient system for further study of SARS-CoV-2 genetic variants and the development of future Kazakhstan’s genomic surveillance program. The SARS-CoV-2 whole-genome was sequenced according to SARS-CoV-2 ARTIC protocol (EXP-MRT001) by Oxford Nanopore Technologies at the National Laboratory Astana, Kazakhstan to track viral variants circulating in the country. The 500 samples kindly provided by the Republican Diagnostic Center (UMC-NU) and private laboratory KDL “Olymp” were collected from individuals in Nur-Sultan city diagnosed with COVID-19 from August 2021 to May 2022 using real-time reverse transcription-quantitative polymerase chain reaction (RT-qPCR). All samples had a cycle threshold (Ct) value below 20 with an average Ct value of 17.03. The overall average value of sequencing depth coverage for samples is 244X. 341 whole-genome sequences that passed quality control were deposited in the Global initiative on sharing all influenza data (GISAID). The BA.1.1 (*n* = 189), BA.1 (*n* = 15), BA.2 (*n* = 3), BA.1.15 (*n* = 1), BA.1.17.2 (*n* = 1) omicron lineages, AY.122 (*n* = 119), B.1.617.2 (*n* = 8), AY.111 (*n* = 2), AY.126 (*n* = 1), AY.4 (*n* = 1) delta lineages, one sample B.1.1.7 (*n* = 1) belongs to alpha lineage, and one sample B.1.637 (*n* = 1) belongs to small sublineage were detected in this study. This is the first study of SARS-CoV-2 whole-genome sequencing by the ONT approach in Kazakhstan, which can be expanded for the investigation of other emerging viral or bacterial infections on the country level.

## Introduction

The global coronavirus disease 2019 (COVID-19) pandemic is caused by severe acute respiratory syndrome coronavirus 2 (SARS-CoV-2) which is responsible for the severe acute respiratory syndrome (Wu et al., 2020; Zhu et al., 2020) that led to the death of 6.3 million people by July 2022 (WHO 2022). Originated in Wuhan, the capital of Hubei province in late December 2019, SARS-CoV-2 immediately propagated across the world causing infectious pneumonia. Primarily, initial clinical symptoms, such as sore throat, fever, weakness, and respiratory distress resembled viral pneumonia. Nevertheless, genomic examination of samples obtained from the infected patients confirmed the novel disease as coronavirus (2019-nCoV) pneumonia (Zhou et al., 2020). Then novel virus was renamed SARS-CoV-2 by the International Committee on Taxonomy of Viruses (ICTV) due to its genetic relevance to earlier verified coronaviruses (Lu et al., 2020). SARS-CoV-2 is a positive-sense single-stranded RNA virus that is composed of approximately 30 kilobase pairs (Wu et al., 2020; Zhu et al., 2020) and has variable open reading frames (ORFs), substantially resembling human coronaviruses (HCoVs) (Song et al., 2019). The viral genome encodes for structural proteins, to be specific nucleocapsid (N), membrane (M), envelope (E), spike (S), sixteen non-structural proteins, namely NSP1-NSP16, and nine accessory proteins - ORF3a, 3d, 6, 7a, 7b, 8, 9b, 14, and 10 (Yadav et al., 2021). Currently, research studies expand understanding of the viral genetics and structural and non-structural proteins that may act as targets of novel drugs for clinical therapeutics (Chellapandi and Saranya, 2020; Shamsi et al., 2021; Yadav et al., 2021). The complete genome sequencing facilitated the advancement of RT-PCR assays for SARS-CoV-2 investigation to standardize the diagnostics of the COVID-19 outbreak (Lu et al., 2020). Viral genome surveillance is critical to monitoring disease transmission during major outbreaks (Gardy et al., 2015). Real-time gene sequencing is a key parameter to manage viral outbreaks because it expands our understanding of the virus proliferation, spread, and evolution (Gardy et al., 2015; Dudas et al., 2017). Primarily, it relies on the prompt sequencing technique of viral genome immediately from clinical specimens without the necessity of viral culturing step (Quick et al., 2017). The Ebola virus epidemic of 2013–2016 demonstrated that viral genome surveillance can yield crucial evidence on Ebola virus progression and facilitate epidemiological examination. SARS-CoV-2 genome sequencing provided important data on the viral mutation rate, transmission dynamics, and its taxonomic origin. Genomic surveillance of SARS-CoV-2 is critical for tracking viral spread in each country, detecting the geographical origin of viral strains, or indication of control measures efficiency, and viral evolution. Besides, the genomic analysis yields vital insights into epidemiological investigations of pandemic evolution. Altogether cumulative investigations facilitated the establishment of nomenclature systems for various SARS-CoV-2 lineages (Rambaut et al., 2020). The disease has been exponentially spreading around the world, and the first SARS-CoV-2 case in Kazakhstan was confirmed to be on the 16th of March 2020, in Almaty city (Zhalmagambetov et al., 2020). To date, by 12 July 2022, over 1.3 million confirmed cases and over 19, 018 deaths have been reported associated with COVID-19 in Kazakhstan (Kazakhstan 2022). As of 11 June 2022, in general, 26 million vaccine doses were administered as reported to WHO (WHO 2022). Although the government implemented several lockdowns, strict quarantine regimes, and restrictions to prevent massive COVID-19 spread, gaps exist in the interpretation of the clinical and epidemiological characterization of the local pandemic. The previous nationwide retrospective cohort study distinguished samples of five of the eight global SARS-CoV-2 clades detected in the early stages of pandemics in Kazakhstan. Besides, it was suggested that a unique lineage (B.4.1) arose independently in Kazakhstan. Genomic surveillance is critical in the representation of the genetic diversity of circulating SARS-CoV-2 that in terms reflects the clinical and epidemiological situation in the country (Yegorov et al., 2021). Besides, the genomic analysis of SARS-CoV-2 variant sequences from Kazakhstan and molecular epidemiology data is expected to facilitate the mapping of viral origin and transmission surveillance (Nidom et al., 2021). As of 12 July 2022, overall, there are 1,325 SARS-CoV-2 genomic variants from Kazakhstan available on GISAID platform, among which only 970 samples are complete genomes, and exclusively Center for Life Sciences, National Laboratory Astana, Nazarbayev University performed whole genome sequencing by ONT in our country. The scarce amount of data on the viral whole genome from Kazakhstan requires the establishment of a functional surveillance program to detect viral mutations. SARS-CoV-2 sequencing was initiated at the National Laboratory of Astana for several reasons, (1) to verify the feasibility of Oxford nanopore amplicon-based SARS-CoV-2 genome sequencing at our institution; and (2) to contribute to SARS-CoV-2 genome surveillance in Nur-Sultan, and (3) to establish an optimized protocol for future SARS-CoV-2 monitoring in Kazakhstan. In this study, the 500 SARS-CoV-2 samples obtained from the RT-PCR confirmed COVID-19 positive patients were sequenced by third-generation sequencing platform Oxford nanopore technology to characterize viral dynamics in the country in connection with the global pandemic.

## Materials and methods

### Sample collection

The 500 samples used in this study were nasopharyngeal swabs kindly provided by the Republican Diagnostic Center (RDC) and private laboratory KDL “Olymp.” The nasopharyngeal swab fluid samples (5–10 ml) were obtained from COVID-19 positive patients whose status was laboratory-confirmed by RT-qPCR results from f August 2021 to May 2022. Viral RNA was isolated from clinical biomaterials using ALPREP extraction kit following manufacturer (Algimed Techno, Belarus) instructions at the RDC laboratory. All samples had a cycle threshold (Ct) value below 20, while the average Ct value of all RNA samples was 17.03 corresponding to a high viral genetic material load.

### ONT library preparation and sequencing

ONT library was prepared according to ARTIC Midnight protocol PCR tiling of SARS-CoV-2 virus with rapid barcoding kit (SQK-RBK110.96) and sequenced on the PromethION48 sequencing platform. The 8 μl RNA samples were reversely transcribed with 2 μl LunaScript RT SuperMix (LS RT) at a thermal cycler using the following program: at 25°C for 2 min, at 55°C for 10 min, at 95°C for 1 min, and at 4°C hold. Midnight RT PCR Expansion (EXP-MRT001) contained separate primer pools (Freed et al., 2020) used for the overlapping tiled PCR reactions spanning the viral genome. The PCR reaction mix for 96 samples contains 241 μl of nuclease-free water, 6 μl of Pool A or Pool B Midnight Primers, and 687 μl of Q5 HS Master Mix (Q5). Two-midnight primer pools were used for annealing 4.5% of the genome and produce 1200 bp amplicons that overlap by approximately 20 bp. The PCR amplification step was carried out under the following conditions: an initial denaturation step at 98°C for 30 s, followed by 35 amplifications at 98°C for 15 s, at 65°C for 5 min, and 4°C holds. The addition of rapid barcodes was performed in the 96-well Barcode Attachment Plate by mixing 2.5 μl nuclease-free water, 5 μl pooled PCR products (from pools A and B), and 2.5 μl barcodes from the Rapid Barcode Plate. The reaction was incubated in a thermal cycler at 30°C for 2 min and then at 80°C for 2 min. A two-step lean-up was performed using the SPRI beads and 80% ethanol. To measure the concentration of DNA (PCR products and DNA libraries), Qubit dsDNA HS Assay Kit was used for a fluorometric measurement of DNA (Thermo Fisher Scientific) on a Qubit 4.0 Fluorometer. The >1400 ng of DNA library was loaded onto a primed PromethION48 flow cell (PAH13359).

### Software setup and installation

The ARTIC sequencing data obtained by the Midnight protocol was analyzed by the wf-artic bioinformatics pipeline. The pipeline was used to prepare and annotate a consensus sequence of every sequenced sample. Wf-artic is managed by Nextflow and is run using Docker (Ewels et al., 2020). The installation of the software on the Linux operating system is straightforward and is supported on GridION and PromethION devices. After the demultiplexing step, the sequence reads were processed by ARTIC FieldBioinformatics software that was adapted to analyze FASTQ Nanopore sequences. Besides, the ARTIC pipeline was modified to utilize a primer scheme that specifies the sequencing primers used in the Midnight protocol and their genomic localization on the SARS-CoV-2 genome. The wf-artic pipeline classifies the sequenced samples according to Nexclade clastidic analysis and Pangolin strain assignment.

### Demultiplexing of multiple barcoded samples

Demultiplexed FASTQ format sequence data is required for the wf-artic workflow. Guppy 6.1.5 performs basecalling of all reads and identifies barcodes in the sequence. To prevent re-basecalling, the software copies the reads pertaining to each barcode to the corresponding tag output directory. Since Midnight protocol utilizes a rapid barcoding kit, the demultiplexing step does not need barcodes at both ends of the sequence. In addition, filtering against mid-strand barcodes is not required.

### Variant calling and phylogenetic profiling

Medaka is a bioinformatics tool that generates consensus sequences from basecalled data by using a collection of individual sequencing reads against a draft assembly. The variant calling was performed by the set of utilities bcftools 1.12. The pipeline output includes NextClade and Pangolin analysis that includes the clade designation according to GISIAD and Pangolin nomenclature. Then consensus sequences were submitted to the GISAID database. Figures were drawn and edited by Nextstrain, BioRender and OriginPro tools.

## Results

**FIGURE 1:**
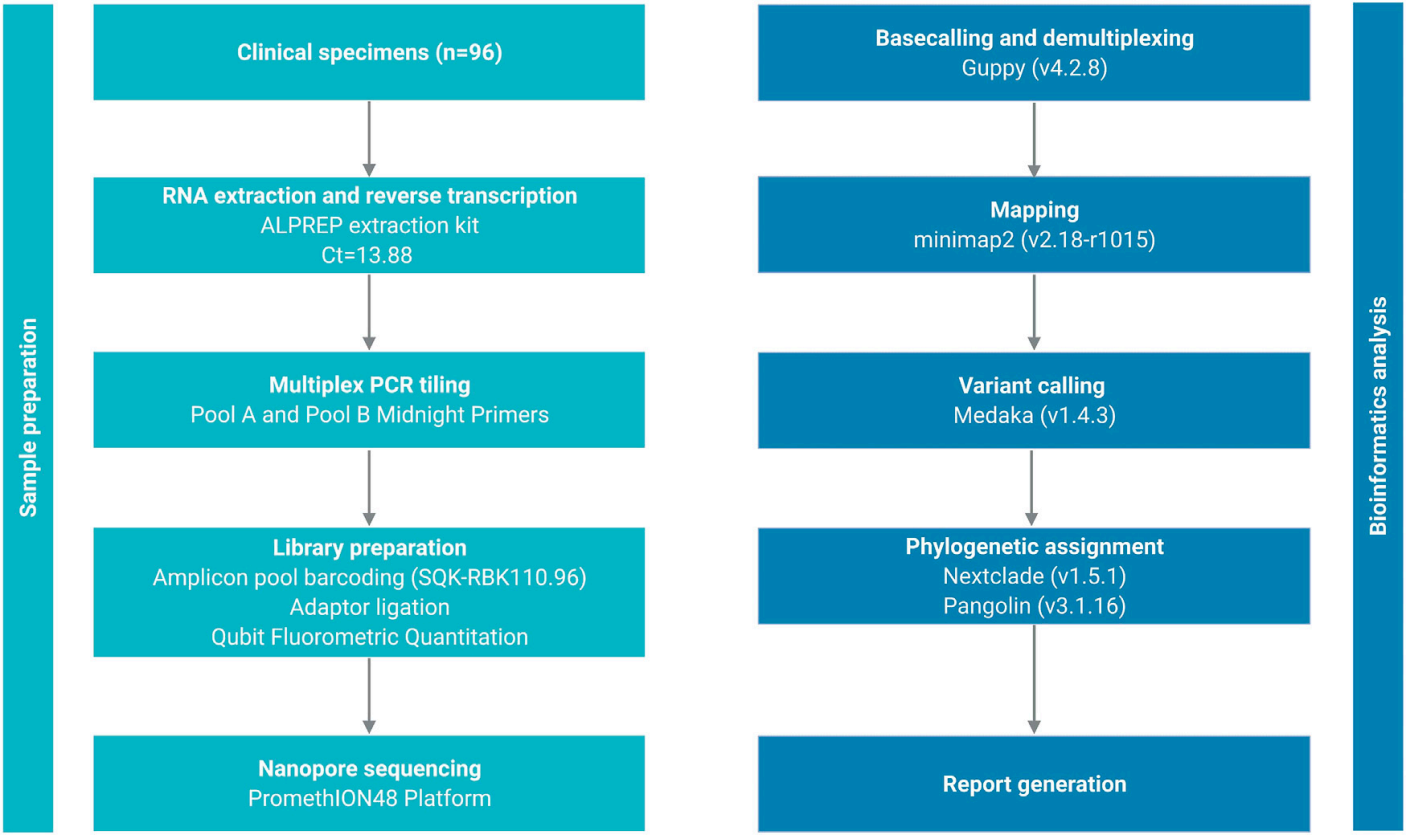
SARS-CoV-2 whole-genome sequencing and analysis workflow.

**FIGURE 2:**
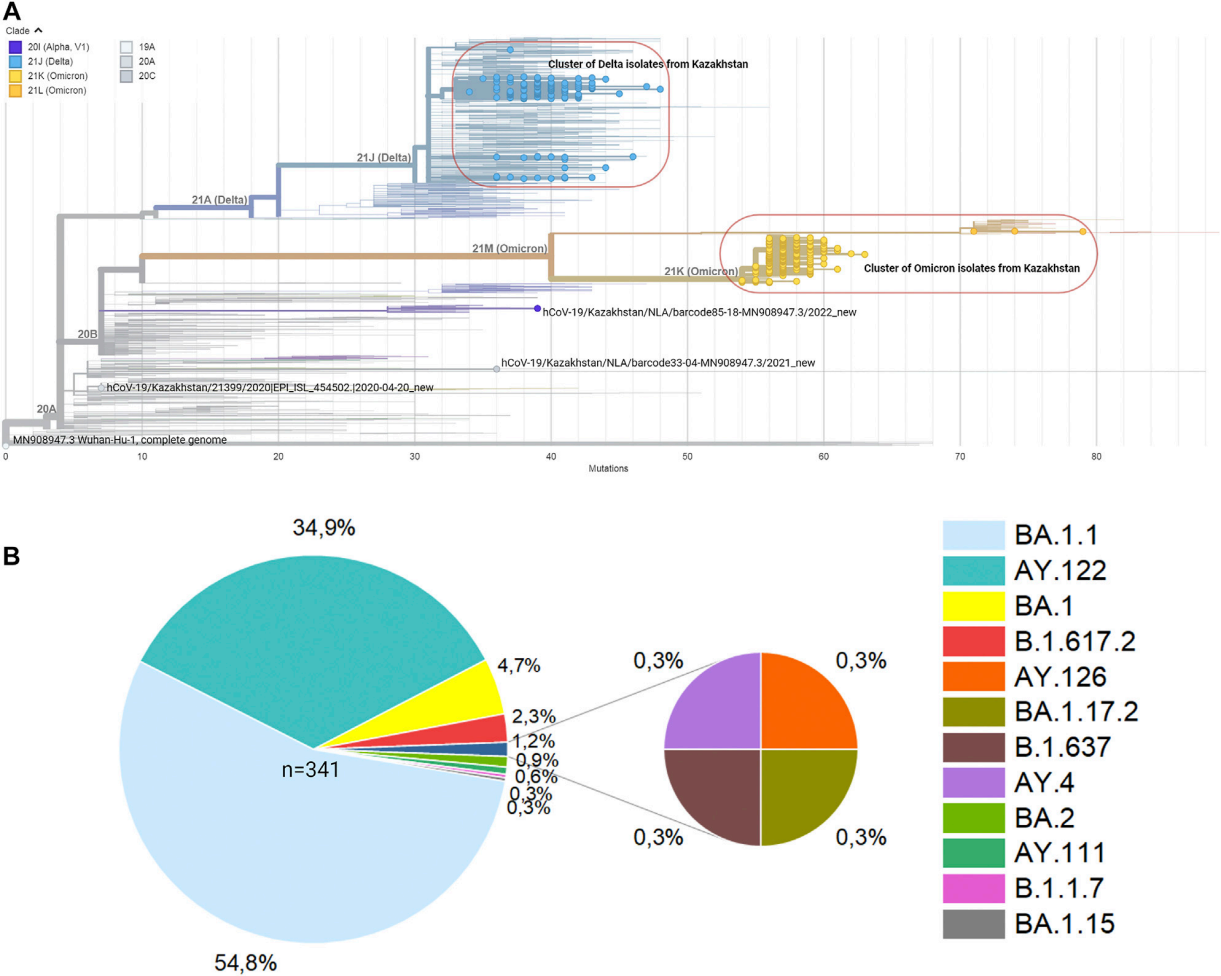
Phylogenetic distribution of sequenced SARS-CoV-2 genomes. **(A)** A phylogenetic tree is generated by Nextstrain. **(B).** The proportion of clades and Pangolin lineages of *n* = 341 sequenced SARS-CoV-2 samples.

**FIGURE 3:**
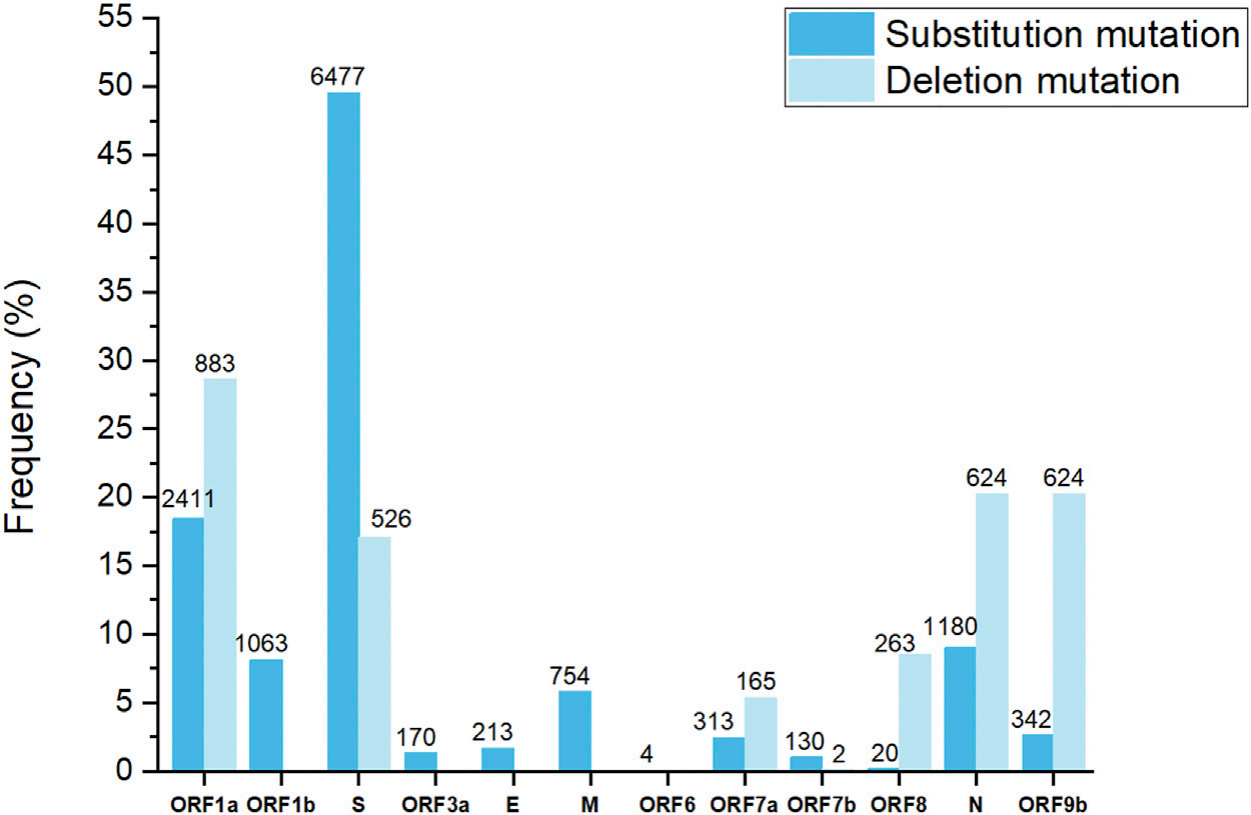
Substitution and deletion mutation frequency in sequenced SARS-CoV-2 genomes. The numbers indicate the number of mutations.

All 500 sample sequences used in this study were obtained from Nur-Sultan from August 2021 to May 2022 and 341 sequences that passed quality control are deposited in the Global initiative on sharing all influenza data (GISAID) (https://www.gisaid.org/hcov19-variants/). SARS-CoV-2 whole-genome sequencing and analysis workflow is outlined in Figure 1. Accession IDs are included in Supplementary Table S1. After successful viral whole-genome sequencing, the obtained data were analyzed according to nCoV-2019 novel coronavirus bioinformatics protocol. Patient and sequenced genome characteristics can be seen in Supplementary Table S2. Most of the sequenced genomes had a GC content of around 38% in the coding sequence which is consistent with previous research studies (Li Y. et al., 2020). The overall average value of sequencing depth coverage is 244 X (Table 1). The amplicon length spanned around 200 to 1100 bp. The low number of reads is associated with low RNA quality and the low-quality reads (*n* = 159) that have more than 3000 missing base pairs were eliminated from the further analysis and the average value of missing nucleotides is equal to 1281. Phylogenetic analysis was performed by uploading 341 sequences from this study and one sample (hCoV-19/Kazakhstan/21399/2020|EPI_ISL_454502|2020-04-20_new) from the previous SAR-CoV-2 study performed in Kazakhstan on Nexstrain open-source toolkit (Yegorov et al., 2021). Lineages were assigned to the sequenced genomes according to the Nextstrain SARS-CoV-2 clades. Out of 341 samples, 205 genomes (60.1%) were clustered under 21K (omicron), 131 genomes (38.4%) were clustered under clade 21J (Delta), 3 genomes (0.9%) were clustered under clade 21L (omicron), 1 genome (0.3%) 20I (Alpha, V1), whereas 1 genome (0.3%) (barcode33 4-MN908947.3_new) was clustered under clade 20C that is a large genetically distinct subclade of 20A that emerged at the beginning of 2020, and the hCoV-19/Kazakhstan/21399/2020|EPI_ISL_454502|2020-04-20 sample that emerged earlier was clustered under 20A branch (Figure 2A). The sequencing analysis of samples from Nur-Sultan city revealed that samples (n = 189) belong to BA.1.1, BA.1 (*n* = 15), BA.2 (*n* = 3), BA.1.15 (*n* = 1), BA.1.17.2 (*n* = 1) according to the Pango lineage or omicron variant. Besides, AY.122 (*n* = 119), B.1.617.2 *(n* = 86), AY.111 (*n* = 2), AY.126 (*n* = 1), and AY.89 (*n* = 1) AY.4 (*n* = 1) lineage samples that belong to delta variants were identified (Figure 2B). All these variants belong to the delta variant according to WHO nomenclature, and GK as designated by GISAID. One B.1.637 sample belonging to a separate lineage corresponding to Iota (original B.1.526) or GH in GISAID nomenclature. Phylogenetic representation suggests that it has higher relevance to the genome hCoV-19/Kazakhstan/21399/2020|EPI_ISL_454502|2020-04-20 due to their earlier emergence in time. Generally, viral whole genome sequencing confirmed that the SARS-CoV-2 variants in the region predominantly belong to delta and omicron strains, correspondingly as a global trend. Essentially, viruses continually evolve as genetic mutations accumulate during the genomic replication step. A lineage is a group of viral variants that evolved from a common progenitor. A variant is a viral group that possesses one or more mutations that distinguish it from other viral variants. Since the emergence of the SARS-CoV-2 in December 2019, it has undergone various mutations that alter its characteristics, such as transmissibility, virulence, antigenicity, and vaccine efficiency (Cosar et al., 2022). Most of the mutations do not facilitate significant alterations in virulence. No novel mutations were detected in the analyzed genomes. The substitution mutations in the sequenced samples particularly occur in S (49.5%) and ORF1a (18.4%), while deletion mutations are mostly found in ORF1a (28.6%), N (20.2%), ORF9b (20.2%) and S (17%) regions (Figure 3). Even though no novel mutations were detected in this study, rare mutations (<100 occurrences in GISAID database) were found in the sequenced samples, specifically, substitution mutations NSP10 V7G, NSP12 S520K, and NSP12 Y38F are the rarest (Supplementary Table S3). NSP10 V7G amino acid substitution was detected twice in two countries: in January 2022, the United States, for the first time (EPI_ISL_9374627), and most recently in April 2022, Israel, (EPI_ISL_12882728). NSP10 plays a key role in viral transcription by inducing NSP14 3′-5′ exoribonuclease and NSP16 2′-O-methyltransferase activities. NSP10 V7G substitution affects a residue that was shown to be important in NSP10-NSP14 interaction, although the substitution is conservative (Lin et al., 2021). NSP12 S520K substitution occurred three times (EPI_ISL_1267723, EPI_ISL_6809575, EPI_ISL_5992397) since February 2021 and the most recently in October 2021. Besides, amino acid change NSP12_Y38F occurred 9 times in the seven countries and the first sample with this mutation was identified in May 2020 (EPI_ISL_11468761) and most recently occurred in June 2022 (EPI_ISL_13576951). NSP12 (RNA-dependent RNA polymerase) is important for viral replication and transcription, however exact effects of the aforementioned mutations are unknown (Kirchdoerfer and Ward 2019).

**TABLE 1:** Average summary characteristics of sequenced SARS-CoV-2 samples by ONT platform.

GC-content (%)	Depth	Ct value	Females	Males	Ns
36.39	243.71	17.03	169	117	1281

## Discussion and conclusion

The documentation confirming the reinfections suggests that distinct SARS-CoV-2 strains can infect the same person (Tillett et al., 2021; To et al., 2021). Genomic sequencing is required to verify these reinfections and eliminate medical recidivism. Rapid and reliable sequencing techniques in the clinical application are crucial for epidemiological supervision (Gonzalez-Recio et al., 2021). Consequently, early reliable detection is crucial in COVID-19 surveillance. Although the antibody-based detection approach is fast, this method has several limitations, specifically, bacterial contamination, hemolysis, fibrin presence, patient autoantibodies, and promoting false-positive results. Accordingly, the sequence detection method remains to be the most appropriate for COVID-19 diagnostics, and viral mutation rate control. In Particular, real-time quantitative reverse transcription-polymerase reaction (RT-qPCR) is the most prominent testing technique for SARS-CoV-2 identification. RT-qPCR is highly specific, fast, and financially affordable, yet it cannot accurately examine amplified gene fragments. Therefore, COVID-19 positive infection is verified by the detection of one or more conservative sites by RT-qPCR. Also, RT-qPCR method possesses a high level of false-negative rates in clinical settings that can cause disease to spread via postponed patient isolation and curing, facilitating further viral transmission (Wang et al., 2020). In association with different sequencing techniques, currently, third-generation sequencing of the SARS-CoV-2 whole genome by Oxford Nanopore Technology is one of the prominent approaches. The main advantages of this platform are long genome reads, an optimized analysis pipeline, rapid data collection (Li J. et al., 2020). Globally, many laboratories are proceeding to optimize the whole-genome sequencing of SARS-CoV-2 in terms of cost and efficiency to benefit epidemiological surveillance as the virus is mutating. As of 12 July 2022, there are only a total of 1,325 viral genomes available on the GISAID platform including 341 sequences from this study submitted from Kazakhstan. Five hundred COVID-19 samples collected in Nur-Sultan, in the period of August 2021 to May 2022 were sequenced by ONT at Center for Life Sciences, National Laboratory Astana, Nazarbayev University. To the best of our knowledge, this is the first study of SARS-CoV-2 whole-genome sequencing by the ONT approach in Kazakhstan. A significant increase in the number of COVID-19 cases confirmed in August 2021 in Kazakhstan facilitated a need to establish an effective scientific and proficient system for further study of SARS-CoV-2 surveillance. To prevent cross-species transmissions and manage outbreaks in the future, healthcare authorities would be able to design measures by understanding the genesis, intervention, and evolutionary process of zoonotic viruses. The phylogenetic tree effectively infers the viral evolution and summarizes the emerging SARS-CoV-2 variants by comparing novel mutations with the query sequence (Fahmi et al., 2021). Integration of genomic and phylogenetic examinations in the evaluation of epidemiological situations in the region would facilitate recognition of risk for viral transmission and the introduction of efficient preventive measures. Further high-throughput analysis and SARS-CoV-2 monitoring in Nur-Sultan city are expected by the GridION ONT sequencer in our Center. A successfully implemented platform of third-generation sequencing by Oxford Nanopore technology showed a cost-effective and rapid approach for the investigation of full spectrum mutations in SARS-CoV-2 samples and may be adopted also for monitoring other viral pandemic outbreaks on the country level. The results of the whole genome sequencing can significantly support the scientific foundation for public health measures, thereby facilitating the improvement of epidemiological situations and increase of public awareness. In conclusion, this SARS-CoV-2 whole-genome sequencing study further characterized the genetic diversity of viral strains and different lineages circulating in Nur-Sultan city, Kazakhstan. Generated and analyzed viral whole-genome data may serve as a reference background for future vaccine construction and comparative genome studies among different regions for rapid tracking of SARS-CoV-2 outbreaks in Kazakhstan and neighboring Central Asian countries.

## Data Availability

The datasets presented in this study can be found in online repositories. The names of the repository/repositories and accession number(s) can be found in the article/Supplementary Material.
