# Supplementary material for: A high scale SARS-CoV-2 profiling by its whole-genome sequencing using Oxford Nanopore Technology in Kazakhstan
Source: Front Genet. 2022 Sep 2;13:906318. doi: 10.3389/fgene.2022.906318 (PMC9479076; doi:10.3389/fgene.2022.906318)
Supplement: Supplementary file 3 [file Table1.DOCX]

***Supplementary Material***

**Table 1. Summary characteristics of sequenced SARS-CoV-2 samples by ONT**

| **#** | **Sample ID** | **GISAID ID** | **Sample collection date** | **Age** | **Gender** | **Ct value** | **Pango Lineage** | **GC-content** | **Depth** | **Ns** |
| --- | --- | --- | --- | --- | --- | --- | --- | --- | --- | --- |
| 1 | hCoV-19/Kazakhstan/NLA/barcode25-01-MN908947.3/2021 | EPI_ISL_13717748 | 2021-09-02 | unknown | unknown | unknown | B.1.617.2 | 0.38 | 321X | 305 |
| 2 | hCoV-19/Kazakhstan/NLA/barcode27-01-MN908947.3/2021 | EPI_ISL_13717749 | 2021-09-02 | unknown | unknown | unknown | AY.122 | 0.38 | 339X | 302 |
| 3 | hCoV-19/Kazakhstan/NLA/barcode28-01-MN908947.3/2021 | EPI_ISL_13717750 | 2021-09-02 | unknown | unknown | unknown | AY.122 | 0.38 | 345X | 275 |
| 4 | hCoV-19/Kazakhstan/NLA/barcode29-01-MN908947.3/2021 | EPI_ISL_13717751 | 2021-09-02 | unknown | unknown | unknown | AY.122 | 0.36 | 296X | 1237 |
| 5 | hCoV-19/Kazakhstan/NLA/barcode31-01-MN908947.3/2021 | EPI_ISL_13717752 | 2021-09-02 | unknown | unknown | unknown | AY.122 | 0.37 | 311X | 704 |
| 6 | hCoV-19/Kazakhstan/NLA/barcode33-01-MN908947.3/2021 | EPI_ISL_13717753 | 2021-09-02 | unknown | unknown | unknown | AY.122 | 0.37 | 326X | 729 |
| 7 | hCoV-19/Kazakhstan/NLA/barcode34-01-MN908947.3/2021 | EPI_ISL_13717754 | 2021-09-02 | unknown | unknown | unknown | AY.122 | 0.38 | 346X | 306 |
| 8 | hCoV-19/Kazakhstan/NLA/barcode35-01-MN908947.3/2021 | EPI_ISL_13717755 | 2021-09-02 | unknown | unknown | unknown | AY.122 | 0.37 | 316X | 448 |
| 9 | hCoV-19/Kazakhstan/NLA/barcode36-01-MN908947.3/2021 | EPI_ISL_13717756 | 2021-09-02 | unknown | unknown | unknown | AY.122 | 0.38 | 341X | 315 |
| 10 | hCoV-19/Kazakhstan/NLA/barcode37-01-MN908947.3/2021 | EPI_ISL_13717757 | 2021-09-02 | unknown | unknown | unknown | AY.122 | 0.37 | 299X | 698 |
| 11 | hCoV-19/Kazakhstan/NLA/barcode39-01-MN908947.3/2021 | EPI_ISL_13717758 | 2021-09-02 | unknown | unknown | unknown | AY.122 | 0.37 | 325X | 700 |
| 12 | hCoV-19/Kazakhstan/NLA/barcode41-01-MN908947.3/2021 | EPI_ISL_13717759 | 2021-09-02 | unknown | unknown | unknown | AY.122 | 0.36 | 293X | 1897 |
| 13 | hCoV-19/Kazakhstan/NLA/barcode42-01-MN908947.3/2021 | EPI_ISL_13717760 | 2021-09-02 | unknown | unknown | unknown | AY.122 | 0.36 | 301X | 1489 |
| 14 | hCoV-19/Kazakhstan/NLA/barcode43-01-MN908947.3/2021 | EPI_ISL_13717761 | 2021-09-02 | unknown | unknown | unknown | AY.122 | 0.38 | 318X | 300 |
| 15 | hCoV-19/Kazakhstan/NLA/barcode45-01-MN908947.3/2021 | EPI_ISL_13717762 | 2021-09-02 | unknown | unknown | unknown | AY.122 | 0.38 | 352X | 289 |
| 16 | hCoV-19/Kazakhstan/NLA/barcode46-01-MN908947.3/2021 | EPI_ISL_13717763 | 2021-09-02 | unknown | unknown | unknown | AY.122 | 0.38 | 339X | 305 |
| 17 | hCoV-19/Kazakhstan/NLA/barcode47-01-MN908947.3/2021 | EPI_ISL_13717764 | 2021-09-02 | unknown | unknown | unknown | AY.122 | 0.37 | 327X | 820 |
| 18 | hCoV-19/Kazakhstan/NLA/barcode02-02-MN908947.3/2021 | EPI_ISL_13717765 | 2021-09-02 | unknown | unknown | unknown | AY.122 | 0.36 | 293X | 1797 |
| 19 | hCoV-19/Kazakhstan/NLA/barcode06-02-MN908947.3/2021 | EPI_ISL_13717766 | 2021-09-02 | unknown | unknown | unknown | AY.122 | 0.35 | 288X | 2669 |
| 20 | hCoV-19/Kazakhstan/NLA/barcode11-02-MN908947.3/2021 | EPI_ISL_13717767 | 2021-09-02 | unknown | unknown | unknown | AY.122 | 0.36 | 324X | 1266 |
| 21 | hCoV-19/Kazakhstan/NLA/barcode20-02-MN908947.3/2021 | EPI_ISL_13717768 | 2021-09-02 | unknown | unknown | unknown | AY.122 | 0.35 | 260X | 2508 |
| 22 | hCoV-19/Kazakhstan/NLA/barcode21-02-MN908947.3/2021 | EPI_ISL_13717769 | 2021-09-02 | unknown | unknown | unknown | AY.122 | 0.36 | 305X | 1347 |
| 23 | hCoV-19/Kazakhstan/NLA/barcode26-02-MN908947.3/2021 | EPI_ISL_13717770 | 2021-09-02 | unknown | unknown | unknown | AY.122 | 0.37 | 320X | 937 |
| 24 | hCoV-19/Kazakhstan/NLA/barcode34-02-MN908947.3/2021 | EPI_ISL_13717771 | 2021-09-02 | unknown | unknown | unknown | AY.122 | 0.36 | 323X | 1862 |
| 25 | hCoV-19/Kazakhstan/NLA/barcode36-02-MN908947.3/2021 | EPI_ISL_13717772 | 2021-09-02 | unknown | unknown | unknown | AY.111 | 0.37 | 326X | 862 |
| 26 | hCoV-19/Kazakhstan/NLA/barcode37-02-MN908947.3/2021 | EPI_ISL_13717773 | 2021-09-02 | unknown | unknown | unknown | AY.122 | 0.38 | 348X | 318 |
| 27 | hCoV-19/Kazakhstan/NLA/barcode39-02-MN908947.3/2021 | EPI_ISL_13717774 | 2021-09-02 | unknown | unknown | unknown | AY.111 | 0.35 | 3X | 2289 |
| 28 | hCoV-19/Kazakhstan/NLA/barcode47-02-MN908947.3/2021 | EPI_ISL_13717775 | 2021-09-02 | unknown | unknown | unknown | AY.122 | 0.36 | 200X | 1789 |
| 29 | hCoV-19/Kazakhstan/NLA/barcode55-02-MN908947.3/2021 | EPI_ISL_13717776 | 2021-09-02 | unknown | unknown | unknown | AY.122 | 0.36 | 124X | 1522 |
| 30 | hCoV-19/Kazakhstan/NLA/barcode56-02-MN908947.3/2021 | EPI_ISL_13717777 | 2021-09-02 | unknown | unknown | unknown | AY.122 | 0.37 | 306X | 368 |
| 31 | hCoV-19/Kazakhstan/NLA/barcode60-02-MN908947.3/2021 | EPI_ISL_13717778 | 2021-09-02 | unknown | unknown | unknown | AY.122 | 0.37 | 243X | 377 |
| 32 | hCoV-19/Kazakhstan/NLA/barcode61-02-MN908947.3/2021 | EPI_ISL_13717779 | 2021-09-02 | unknown | unknown | unknown | AY.122 | 0.38 | 3X | 328 |
| 33 | hCoV-19/Kazakhstan/NLA/barcode62-02-MN908947.3/2021 | EPI_ISL_13717780 | 2021-09-02 | unknown | unknown | unknown | AY.122 | 0.38 | 335X | 337 |
| 34 | hCoV-19/Kazakhstan/NLA/barcode63-02-MN908947.3/2021 | EPI_ISL_13717781 | 2021-09-02 | unknown | unknown | unknown | AY.122 | 0.38 | 347X | 321 |
| 35 | hCoV-19/Kazakhstan/NLA/barcode64-02-MN908947.3/2021 | EPI_ISL_13717782 | 2021-09-02 | unknown | unknown | unknown | AY.122 | 0.38 | 344X | 353 |
| 36 | hCoV-19/Kazakhstan/NLA/barcode66-02-MN908947.3/2021 | EPI_ISL_13717783 | 2021-09-02 | unknown | unknown | unknown | AY.122 | 0.37 | 319X | 854 |
| 37 | hCoV-19/Kazakhstan/NLA/barcode68-02-MN908947.3/2021 | EPI_ISL_13717784 | 2021-09-02 | unknown | unknown | unknown | AY.122 | 0.34 | 304X | 2851 |
| 38 | hCoV-19/Kazakhstan/NLA/barcode69-02-MN908947.3/2021 | EPI_ISL_13717785 | 2021-09-02 | unknown | unknown | unknown | AY.122 | 0.37 | 128X | 449 |
| 39 | hCoV-19/Kazakhstan/NLA/barcode71-02-MN908947.3/2021 | EPI_ISL_13717786 | 2021-09-02 | unknown | unknown | unknown | AY.122 | 0.37 | 279X | 349 |
| 40 | hCoV-19/Kazakhstan/NLA/barcode72-02-MN908947.3/2021 | EPI_ISL_13717787 | 2021-09-02 | unknown | unknown | unknown | B.1.617.2 | 0.36 | 31X | 1732 |
| 41 | hCoV-19/Kazakhstan/NLA/barcode75-02-MN908947.3/2021 | EPI_ISL_13717788 | 2021-09-02 | unknown | unknown | unknown | AY.122 | 0.35 | 200X | 2243 |
| 42 | hCoV-19/Kazakhstan/NLA/barcode76-02-MN908947.3/2021 | EPI_ISL_13717789 | 2021-09-02 | unknown | unknown | unknown | AY.4 | 0.37 | 249X | 1015 |
| 43 | hCoV-19/Kazakhstan/NLA/barcode81-02-MN908947.3/2021 | EPI_ISL_13717790 | 2021-09-02 | unknown | unknown | unknown | AY.122 | 0.36 | 167X | 1300 |
| 44 | hCoV-19/Kazakhstan/NLA/barcode82-02-MN908947.3/2021 | EPI_ISL_13717791 | 2021-09-02 | unknown | unknown | unknown | AY.122 | 0.37 | 221X | 562 |
| 45 | hCoV-19/Kazakhstan/NLA/barcode86-02-MN908947.3/2021 | EPI_ISL_13717792 | 2021-09-02 | unknown | unknown | unknown | AY.122 | 0.37 | 159X | 534 |
| 46 | hCoV-19/Kazakhstan/NLA/barcode88-02-MN908947.3/2021 | EPI_ISL_13717793 | 2021-09-02 | unknown | unknown | unknown | AY.122 | 0.36 | 302X | 1599 |
| 47 | hCoV-19/Kazakhstan/NLA/barcode89-02-MN908947.3/2021 | EPI_ISL_13717794 | 2021-09-02 | unknown | unknown | unknown | AY.122 | 0.34 | 183X | 2997 |
| 48 | hCoV-19/Kazakhstan/NLA/barcode19-03-MN908947.3/2021 | EPI_ISL_13717795 | 2021-09-02 | unknown | unknown | unknown | AY.122 | 0.35 | 234X | 2325 |
| 49 | hCoV-19/Kazakhstan/NLA/barcode71-03-MN908947.3/2021 | EPI_ISL_13717796 | 2021-09-02 | unknown | unknown | unknown | AY.122 | 0.35 | 213X | 1939 |
| 50 | hCoV-19/Kazakhstan/NLA/barcode79-03-MN908947.3/2021 | EPI_ISL_13717797 | 2021-09-02 | unknown | unknown | unknown | AY.122 | 0.35 | 249X | 2496 |
| 51 | hCoV-19/Kazakhstan/NLA/barcode87-03-MN908947.3/2021 | EPI_ISL_13717798 | 2021-09-02 | unknown | unknown | unknown | AY.122 | 0.35 | 231X | 2562 |
| 52 | hCoV-19/Kazakhstan/NLA/barcode01-04-MN9089047.3/2021 | EPI_ISL_5465367 | 2021-08-02 | 40 | female | 15.24 | B.1.617.2 | 0.38 | 372X | 316 |
| 53 | hCoV-19/Kazakhstan/NLA/barcode02-04-MN9089047.3/2021 | EPI_ISL_5465373 | 2021-08-02 | 42 | male | 11.89 | B.1.617.2 | 0.38 | 374X | 314 |
| 54 | hCoV-19/Kazakhstan/NLA/barcode03-04-MN9089047.3/2021 | EPI_ISL_5465381 | 2021-08-02 | 33 | male | 14.87 | B.1.617.2 | 0.38 | 360X | 315 |
| 55 | hCoV-19/Kazakhstan/NLA/barcode04-04-MN9089047.3/2021 | EPI_ISL_5465387 | 2021-08-02 | 22 | female | 13.97 | B.1.617.2 | 0.39 | 367X | 314 |
| 56 | hCoV-19/Kazakhstan/NLA/barcode05-04-MN9089047.3/2021 | EPI_ISL_5465398 | 2021-08-02 | 35 | male | 13.45 | B.1.617.2 | 0.38 | 362X | 312 |
| 57 | hCoV-19/Kazakhstan/NLA/barcode06-04-MN9089047.3/2021 | EPI_ISL_5465404 | 2021-08-02 | 24 | male | 13.18 | B.1.617.2 | 0.38 | 372X | 313 |
| 58 | hCoV-19/Kazakhstan/NLA/barcode07-04-MN9089047.3/2021 | EPI_ISL_5465410 | 2021-08-02 | 52 | female | 17.54 | B.1.617.2 | 0.38 | 365X | 314 |
| 59 | hCoV-19/Kazakhstan/NLA/barcode08-04-MN9089047.3/2021 | EPI_ISL_5465417 | 2021-08-02 | 59 | male | 13.05 | B.1.617.2 | 0.38 | 351X | 315 |
| 60 | hCoV-19/Kazakhstan/NLA/barcode09-04-MN9089047.3/2021 | EPI_ISL_5465422 | 2021-08-02 | 32 | female | 14.91 | B.1.617.2 | 0.38 | 369X | 314 |
| 61 | hCoV-19/Kazakhstan/NLA/barcode10-04-MN9089047.3/2021 | EPI_ISL_5465425 | 2021-08-02 | 35 | female | 11.79 | B.1.617.2 | 0.38 | 369X | 315 |
| 62 | hCoV-19/Kazakhstan/NLA/barcode11-04-MN9089047.3/2021 | EPI_ISL_5465431 | 2021-08-02 | 61 | male | 14.72 | B.1.617.2 | 0.38 | 361X | 314 |
| 63 | hCoV-19/Kazakhstan/NLA/barcode12-04-MN9089047.3/2021 | EPI_ISL_5465438 | 2021-08-02 | 28 | male | 19.1 | B.1.617.2 | 0.39 | 352X | 306 |
| 64 | hCoV-19/Kazakhstan/NLA/barcode13-04-MN9089047.3/2021 | EPI_ISL_5465445 | 2021-08-02 | 17 | male | 13.13 | B.1.617.2 | 0.38 | 367X | 314 |
| 65 | hCoV-19/Kazakhstan/NLA/barcode14-04-MN9089047.3/2021 | EPI_ISL_5465452 | 2021-08-02 | 33 | male | 8.57 | B.1.617.2 | 0.38 | 368X | 314 |
| 66 | hCoV-19/Kazakhstan/NLA/barcode15-04-MN9089047.3/2021 | EPI_ISL_5465456 | 2021-08-02 | 23 | male | 17.83 | B.1.617.2 | 0.38 | 347X | 315 |
| 67 | hCoV-19/Kazakhstan/NLA/barcode16-04-MN9089047.3/2021 | EPI_ISL_5465463 | 2021-08-02 | 55 | female | 11.65 | B.1.617.2 | 0.37 | 371X | 314 |
| 68 | hCoV-19/Kazakhstan/NLA/barcode17-04-MN9089047.3/2021 | EPI_ISL_5465470 | 2021-08-02 | 36 | male | 15.56 | B.1.617.2 | 0.38 | 363X | 314 |
| 69 | hCoV-19/Kazakhstan/NLA/barcode18-04-MN9089047.3/2021 | EPI_ISL_5465480 | 2021-08-02 | 27 | male | 10.76 | B.1.617.2 | 0.38 | 352X | 318 |
| 70 | hCoV-19/Kazakhstan/NLA/barcode19-04-MN9089047.3/2021 | EPI_ISL_5465482 | 2021-08-02 | 27 | male | 16.3 | B.1.617.2 | 0.38 | 356X | 309 |
| 71 | hCoV-19/Kazakhstan/NLA/barcode20-04-MN9089047.3/2021 | EPI_ISL_5465491 | 2021-08-03 | 62 | male | 10.15 | B.1.617.2 | 0.38 | 370X | 315 |
| 72 | hCoV-19/Kazakhstan/NLA/barcode21-04-MN9089047.3/2021 | EPI_ISL_5465494 | 2021-08-03 | 41 | female | 13.58 | B.1.617.2 | 0.38 | 360X | 314 |
| 73 | hCoV-19/Kazakhstan/NLA/barcode22-04-MN9089047.3/2021 | EPI_ISL_5465502 | 2021-08-03 | 28 | female | 13.63 | B.1.617.2 | 0.38 | 368X | 314 |
| 74 | hCoV-19/Kazakhstan/NLA/barcode23-04-MN9089047.3/2021 | EPI_ISL_5465505 | 2021-08-03 | 53 | female | 11.51 | B.1.617.2 | 0.38 | 371X | 314 |
| 75 | hCoV-19/Kazakhstan/NLA/barcode24-04-MN9089047.3/2021 | EPI_ISL_5465507 | 2021-08-03 | 32 | female | 12.85 | B.1.617.2 | 0.37 | 322X | 565 |
| 76 | hCoV-19/Kazakhstan/NLA/barcode25-04-MN9089047.3/2021 | EPI_ISL_5465514 | 2021-08-03 | 31 | male | 9.92 | B.1.617.2 | 0.38 | 372X | 314 |
| 77 | hCoV-19/Kazakhstan/NLA/barcode26-04-MN9089047.3/2021 | EPI_ISL_5465518 | 2021-08-03 | 32 | female | 6.48 | B.1.617.2 | 0.38 | 317X | 333 |
| 78 | hCoV-19/Kazakhstan/NLA/barcode27-04-MN9089047.3/2021 | EPI_ISL_5465523 | 2021-08-03 | 60 | female | 17.87 | B.1.617.2 | 0.38 | 357X | 316 |
| 79 | hCoV-19/Kazakhstan/NLA/barcode28-04-MN9089047.3/2021 | EPI_ISL_5465533 | 2021-08-03 | 31 | male | 17.85 | B.1.617.2 | 0.38 | 362X | 316 |
| 80 | hCoV-19/Kazakhstan/NLA/barcode29-04-MN9089047.3/2021 | EPI_ISL_5532919 | 2021-08-03 | 51 | male | 16.55 | B.1.617.2 | 0.38 | 367X | 314 |
| 81 | hCoV-19/Kazakhstan/NLA/barcode30-04-MN9089047.3/2021 | EPI_ISL_5465539 | 2021-08-03 | 44 | male | 13.94 | B.1.617.2 | 0.38 | 357X | 310 |
| 82 | hCoV-19/Kazakhstan/NLA/barcode31-04-MN9089047.3/2021 | EPI_ISL_5465542 | 2021-08-03 | 48 | female | 17.94 | B.1.617.2 | 0.38 | 358X | 320 |
| 83 | hCoV-19/Kazakhstan/NLA/barcode33-04-MN9089047.3/2021 | EPI_ISL_5465549 | 2021-08-04 | 23 | male | 14.27 | B.1.637 | 0.38 | 362X | 315 |
| 84 | hCoV-19/Kazakhstan/NLA/barcode34-04-MN9089047.3/2021 | EPI_ISL_5465553 | 2021-08-04 | 76 | male | 10.85 | B.1.617.2 | 0.38 | 369X | 314 |
| 85 | hCoV-19/Kazakhstan/NLA/barcode35-04-MN9089047.3/2021 | EPI_ISL_5465563 | 2021-08-04 | 30 | female | 10.09 | B.1.617.2 | 0.38 | 365X | 315 |
| 86 | hCoV-19/Kazakhstan/NLA/barcode36-04-MN9089047.3/2021 | EPI_ISL_5465570 | 2021-08-04 | 27 | male | 10.19 | B.1.617.2 | 0.39 | 370X | 314 |
| 87 | hCoV-19/Kazakhstan/NLA/barcode37-04-MN9089047.3/2021 | EPI_ISL_5465574 | 2021-08-04 | 28 | female | 7.27 | B.1.617.2 | 0.38 | 370X | 304 |
| 88 | hCoV-19/Kazakhstan/NLA/barcode38-04-MN9089047.3/2021 | EPI_ISL_5465584 | 2021-08-04 | 57 | male | 15.01 | AY.39 | 0.38 | 315X | 553 |
| 89 | hCoV-19/Kazakhstan/NLA/barcode39-04-MN9089047.3/2021 | EPI_ISL_5465593 | 2021-08-04 | 38 | male | 11.07 | B.1.617.2 | 0.38 | 373X | 309 |
| 90 | hCoV-19/Kazakhstan/NLA/barcode41-04-MN9089047.3/2021 | EPI_ISL_5465598 | 2021-08-04 | 60 | female | 12.35 | B.1.617.2 | 0.38 | 358X | 323 |
| 91 | hCoV-19/Kazakhstan/NLA/barcode42-04-MN9089047.3/2021 | EPI_ISL_5465603 | 2021-08-04 | 27 | female | 11.08 | B.1.617.2 | 0.38 | 358X | 326 |
| 92 | hCoV-19/Kazakhstan/NLA/barcode43-04-MN9089047.3/2021 | EPI_ISL_5465608 | 2021-08-04 | 27 | male | 17.76 | B.1.617.2 | 0.37 | 338X | 315 |
| 93 | hCoV-19/Kazakhstan/NLA/barcode44-04-MN9089047.3/2021 | EPI_ISL_5465615 | 2021-08-04 | 22 | male | 16.42 | B.1.617.2 | 0.38 | 363X | 314 |
| 94 | hCoV-19/Kazakhstan/NLA/barcode45-04-MN9089047.3/2021 | EPI_ISL_5465620 | 2021-08-04 | 27 | female | 12.05 | B.1.617.2 | 0.38 | 364X | 314 |
| 95 | hCoV-19/Kazakhstan/NLA/barcode46-04-MN9089047.3/2021 | EPI_ISL_5465627 | 2021-08-05 | 48 | female | 18.69 | B.1.617.2 | 0.38 | 347X | 310 |
| 96 | hCoV-19/Kazakhstan/NLA/barcode47-04-MN9089047.3/2021 | EPI_ISL_5465636 | 2021-08-05 | 56 | female | 7.38 | B.1.617.2 | 0.38 | 369X | 328 |
| 97 | hCoV-19/Kazakhstan/NLA/barcode49-04-MN9089047.3/2021 | EPI_ISL_5465643 | 2021-08-05 | 44 | male | 10.81 | B.1.617.2 | 0.39 | 371X | 308 |
| 98 | hCoV-19/Kazakhstan/NLA/barcode50-04-MN9089047.3/2021 | EPI_ISL_5465647 | 2021-08-05 | 37 | male | 12.59 | B.1.617.2 | 0.38 | 348X | 316 |
| 99 | hCoV-19/Kazakhstan/NLA/barcode51-04-MN9089047.3/2021 | EPI_ISL_5465654 | 2021-08-05 | 39 | female | 15.67 | B.1.617.2 | 0.38 | 363X | 320 |
| 100 | hCoV-19/Kazakhstan/NLA/barcode52-04-MN9089047.3/2021 | EPI_ISL_5465658 | 2021-08-05 | 35 | male | 10.63 | B.1.617.2 | 0.38 | 360X | 314 |
| 101 | hCoV-19/Kazakhstan/NLA/barcode53-04-MN9089047.3/2021 | EPI_ISL_5465664 | 2021-08-05 | 24 | female | 14.1 | B.1.617.2 | 0.39 | 367X | 314 |
| 102 | hCoV-19/Kazakhstan/NLA/barcode55-04-MN9089047.3/2021 | EPI_ISL_5532920 | 2021-08-06 | 26 | female | 17.09 | AY.47 | 0.38 | 370X | 314 |
| 103 | hCoV-19/Kazakhstan/NLA/barcode57-04-MN9089047.3/2021 | EPI_ISL_5465668 | 2021-08-06 | 42 | female | 16.18 | B.1.617.2 | 0.38 | 352X | 318 |
| 104 | hCoV-19/Kazakhstan/NLA/barcode58-04-MN9089047.3/2021 | EPI_ISL_5465675 | 2021-08-06 | 37 | male | 17.1 | B.1.617.2 | 0.38 | 338X | 313 |
| 105 | hCoV-19/Kazakhstan/NLA/barcode59-04-MN9089047.3/2021 | EPI_ISL_5465681 | 2021-08-06 | 59 | male | 13.61 | B.1.617.2 | 0.38 | 371X | 314 |
| 106 | hCoV-19/Kazakhstan/NLA/barcode60-04-MN9089047.3/2021 | EPI_ISL_5465687 | 2021-08-06 | 31 | male | 19.65 | B.1.617.2 | 0.38 | 360X | 316 |
| 107 | hCoV-19/Kazakhstan/NLA/barcode61-04-MN9089047.3/2021 | EPI_ISL_5465694 | 2021-08-06 | 41 | female | 13.03 | B.1.617.2 | 0.38 | 366X | 314 |
| 108 | hCoV-19/Kazakhstan/NLA/barcode62-04-MN9089047.3/2021 | EPI_ISL_5465699 | 2021-08-06 | 26 | female | 11.16 | B.1.617.2 | 0.38 | 368X | 309 |
| 109 | hCoV-19/Kazakhstan/NLA/barcode63-04-MN9089047.3/2021 | EPI_ISL_5465707 | 2021-08-06 | 53 | female | 15.44 | B.1.617.2 | 0.38 | 368X | 304 |
| 110 | hCoV-19/Kazakhstan/NLA/barcode65-04-MN9089047.3/2021 | EPI_ISL_5465713 | 2021-08-06 | 33 | male | 11.01 | B.1.617.2 | 0.39 | 365X | 315 |
| 111 | hCoV-19/Kazakhstan/NLA/barcode66-04-MN9089047.3/2021 | EPI_ISL_5465722 | 2021-08-06 | 33 | male | 10.91 | B.1.617.2 | 0.38 | 366X | 315 |
| 112 | hCoV-19/Kazakhstan/NLA/barcode67-04-MN9089047.3/2021 | EPI_ISL_5465730 | 2021-08-06 | 43 | female | 17.98 | B.1.617.2 | 0.37 | 354X | 316 |
| 113 | hCoV-19/Kazakhstan/NLA/barcode68-04-MN9089047.3/2021 | EPI_ISL_5532921 | 2021-08-07 | 34 | female | 17.73 | B.1.617.2 | 0.39 | 342X | 327 |
| 114 | hCoV-19/Kazakhstan/NLA/barcode69-04-MN9089047.3/2021 | EPI_ISL_5465739 | 2021-08-07 | 32 | female | 16.57 | B.1.617.2 | 0.38 | 348X | 315 |
| 115 | hCoV-19/Kazakhstan/NLA/barcode70-04-MN9089047.3/2021 | EPI_ISL_5465744 | 2021-08-07 | 38 | female | 12.53 | B.1.617.2 | 0.38 | 361X | 314 |
| 116 | hCoV-19/Kazakhstan/NLA/barcode71-04-MN9089047.3/2021 | EPI_ISL_5532922 | 2021-08-07 | 36 | male | 17.34 | B.1.617.2 | 0.37 | 354X | 325 |
| 117 | hCoV-19/Kazakhstan/NLA/barcode73-04-MN9089047.3/2021 | EPI_ISL_5532923 | 2021-08-07 | 34 | female | 19.98 | AY.47 | 0.39 | 323X | 324 |
| 118 | hCoV-19/Kazakhstan/NLA/barcode74-04-MN9089047.3/2021 | EPI_ISL_5465751 | 2021-08-07 | 28 | male | 11.51 | AY.39 | 0.38 | 364X | 322 |
| 119 | hCoV-19/Kazakhstan/NLA/barcode75-04-MN9089047.3/2021 | EPI_ISL_5532924 | 2021-08-07 | 16 | female | 11.92 | B.1.617.2 | 0.38 | 368X | 314 |
| 120 | hCoV-19/Kazakhstan/NLA/barcode76-04-MN9089047.3/2021 | EPI_ISL_5465755 | 2021-08-09 | 48 | female | 10.9 | B.1.617.2 | 0.38 | 350X | 320 |
| 121 | hCoV-19/Kazakhstan/NLA/barcode77-04-MN9089047.3/2021 | EPI_ISL_5465759 | 2021-08-09 | 38 | female | 14.54 | B.1.617.2 | 0.38 | 353X | 317 |
| 122 | hCoV-19/Kazakhstan/NLA/barcode78-04-MN9089047.3/2021 | EPI_ISL_5465764 | 2021-08-09 | 46 | female | 14.09 | B.1.617.2 | 0.38 | 365X | 314 |
| 123 | hCoV-19/Kazakhstan/NLA/barcode79-04-MN9089047.3/2021 | EPI_ISL_5465770 | 2021-08-09 | 31 | female | 15.6 | B.1.617.2 | 0.38 | 368X | 320 |
| 124 | hCoV-19/Kazakhstan/NLA/barcode80-04-MN9089047.3/2021 | EPI_ISL_5532925 | 2021-08-09 | 51 | female | 14.32 | B.1.617.2 | 0.37 | 354X | 315 |
| 125 | hCoV-19/Kazakhstan/NLA/barcode81-04-MN9089047.3/2021 | EPI_ISL_5465778 | 2021-08-09 | 27 | male | 14.98 | B.1.617.2 | 0.38 | 354X | 314 |
| 126 | hCoV-19/Kazakhstan/NLA/barcode82-04-MN9089047.3/2021 | EPI_ISL_5465785 | 2021-08-09 | 42 | female | 15.61 | B.1.617.2 | 0.38 | 355X | 315 |
| 127 | hCoV-19/Kazakhstan/NLA/barcode83-04-MN9089047.3/2021 | EPI_ISL_5465790 | 2021-08-09 | 19 | female | 18.13 | B.1.617.2 | 0.38 | 362X | 314 |
| 128 | hCoV-19/Kazakhstan/NLA/barcode84-04-MN9089047.3/2021 | EPI_ISL_5465795 | 2021-08-09 | 34 | female | 12.06 | B.1.617.2 | 0.38 | 370X | 314 |
| 129 | hCoV-19/Kazakhstan/NLA/barcode03-08-MN908947.3/2022 | EPI_ISL_13717799 | 2022-01-06 | 31 | Male | 14.09 | BA.1.1 | 0.36 | 271X | 1266 |
| 130 | hCoV-19/Kazakhstan/NLA/barcode18-08-MN908947.3/2022 | EPI_ISL_13717800 | 2022-01-11 | 52 | Male | 13.56 | BA.1.17.2 | 0.37 | 289X | 778 |
| 131 | hCoV-19/Kazakhstan/NLA/barcode19-08-MN908947.3/2022 | EPI_ISL_13717801 | 2022-01-11 | 21 | Female | 12.7 | BA.1.1 | 0.36 | 269X | 1448 |
| 132 | hCoV-19/Kazakhstan/NLA/barcode25-08-MN908947.3/2022 | EPI_ISL_13717802 | 2022-01-11 | 10 months | Male | 14.84 | BA.1.1 | 0.34 | 263X | 2892 |
| 133 | hCoV-19/Kazakhstan/NLA/barcode26-08-MN908947.3/2022 | EPI_ISL_13717803 | 2022-01-11 | 41 | Female | 13.22 | BA.1.1 | 0.35 | 263X | 2047 |
| 134 | hCoV-19/Kazakhstan/NLA/barcode85-08-MN908947.3/2022 | EPI_ISL_13717804 | 2022-01-22 | 28 | Male | 13.13 | BA.1.1 | 0.35 | 272X | 2232 |
| 135 | hCoV-19/Kazakhstan/NLA/barcode01-10-MN908947.3/2022 | EPI_ISL_13717805 | 2022-01-05 | 59 | Female | 17.24 | BA.1.1 | 0.36 | 297X | 1256 |
| 136 | hCoV-19/Kazakhstan/NLA/barcode07-10-MN908947.3/2022 | EPI_ISL_13717806 | 2022-01-05 | 39 | Female | 18.95 | BA.1.1 | 0.35 | 262X | 2045 |
| 137 | hCoV-19/Kazakhstan/NLA/barcode09-10-MN908947.3/2022 | EPI_ISL_13717807 | 2022-01-05 | 29 | Female | 15.95 | BA.1.1 | 0.37 | 300X | 1167 |
| 138 | hCoV-19/Kazakhstan/NLA/barcode10-10-MN908947.3/2022 | EPI_ISL_13717808 | 2022-01-05 | 35 | Female | 14.82 | BA.1.1 | 0.36 | 219X | 1340 |
| 139 | hCoV-19/Kazakhstan/NLA/barcode14-10-MN908947.3/2022 | EPI_ISL_13717809 | 2022-01-05 | 65 | Female | 13.41 | BA.1 | 0.36 | 250X | 1346 |
| 140 | hCoV-19/Kazakhstan/NLA/barcode17-10-MN908947.3/2022 | EPI_ISL_13717810 | 2022-01-06 | 35 | Male | 20.82 | BA.1.1 | 0.36 | 313X | 1262 |
| 141 | hCoV-19/Kazakhstan/NLA/barcode19-10-MN908947.3/2022 | EPI_ISL_13717811 | 2022-01-06 | 38 | Male | 18.05 | BA.1.1 | 0.36 | 299X | 1467 |
| 142 | hCoV-19/Kazakhstan/NLA/barcode20-10-MN908947.3/2022 | EPI_ISL_13717812 | 2022-01-06 | 18 | Female | 13.69 | BA.1.1 | 0.37 | 308X | 1172 |
| 143 | hCoV-19/Kazakhstan/NLA/barcode21-10-MN908947.3/2022 | EPI_ISL_13717813 | 2022-01-06 | 12 | Female | 18.98 | BA.1.1 | 0.36 | 291X | 1274 |
| 144 | hCoV-19/Kazakhstan/NLA/barcode25-10-MN908947.3/2022 | EPI_ISL_13717814 | 2022-01-06 | 26 | Male | 18.1 | AY.122 | 0.38 | 342X | 315 |
| 145 | hCoV-19/Kazakhstan/NLA/barcode26-10-MN908947.3/2022 | EPI_ISL_13717815 | 2022-01-06 | 62 | Female | 18.98 | BA.1.1 | 0.36 | 312X | 1236 |
| 146 | hCoV-19/Kazakhstan/NLA/barcode27-10-MN908947.3/2022 | EPI_ISL_13717816 | 2022-01-06 | 38 | Female | 13.51 | BA.1.1 | 0.37 | 327X | 990 |
| 147 | hCoV-19/Kazakhstan/NLA/barcode28-10-MN908947.3/2022 | EPI_ISL_13717817 | 2022-01-06 | 28 | Female | 17.85 | BA.1.1 | 0.36 | 297X | 1939 |
| 148 | hCoV-19/Kazakhstan/NLA/barcode29-10-MN908947.3/2022 | EPI_ISL_13717818 | 2022-01-06 | 45 | Female | 19.95 | BA.1.1 | 0.36 | 279X | 1477 |
| 149 | hCoV-19/Kazakhstan/NLA/barcode31-10-MN908947.3/2022 | EPI_ISL_13717819 | 2022-01-08 | 59 | Female | 21.86 | BA.1.1 | 0.35 | 237X | 2196 |
| 150 | hCoV-19/Kazakhstan/NLA/barcode32-10-MN908947.3/2022 | EPI_ISL_13717820 | 2022-01-08 | 38 | Male | 15.36 | BA.1 | 0.36 | 186X | 1243 |
| 151 | hCoV-19/Kazakhstan/NLA/barcode34-10-MN908947.3/2022 | EPI_ISL_13717821 | 2022-01-08 | 57 | Female | 21.53 | BA.1.1 | 0.34 | 237X | 2971 |
| 152 | hCoV-19/Kazakhstan/NLA/barcode37-10-MN908947.3/2022 | EPI_ISL_13717822 | 2022-01-08 | 46 | Female | 16.43 | BA.1.1 | 0.35 | 286X | 2028 |
| 153 | hCoV-19/Kazakhstan/NLA/barcode39-10-MN908947.3/2022 | EPI_ISL_13717823 | 2022-01-08 | 42 | Female | 17.07 | BA.1.1 | 0.36 | 292X | 1269 |
| 154 | hCoV-19/Kazakhstan/NLA/barcode40-10-MN908947.3/2022 | EPI_ISL_13717824 | 2022-01-08 | 34 | Female | 17.76 | BA.1.1 | 0.36 | 273X | 1253 |
| 155 | hCoV-19/Kazakhstan/NLA/barcode41-10-MN908947.3/2022 | EPI_ISL_13717825 | 2022-01-08 | 24 | Female | 21.36 | BA.1.1 | 0.35 | 276X | 2222 |
| 156 | hCoV-19/Kazakhstan/NLA/barcode42-10-MN908947.3/2022 | EPI_ISL_13717826 | 2022-01-08 | 61 | Female | 19.55 | BA.1.1 | 0.36 | 259X | 1941 |
| 157 | hCoV-19/Kazakhstan/NLA/barcode43-10-MN908947.3/2022 | EPI_ISL_13717827 | 2022-01-08 | 28 | Female | 17.25 | BA.1 | 0.38 | 321X | 315 |
| 158 | hCoV-19/Kazakhstan/NLA/barcode44-10-MN908947.3/2022 | EPI_ISL_13717828 | 2022-01-08 | 22 | Female | 19.14 | BA.1.1 | 0.36 | 290X | 1266 |
| 159 | hCoV-19/Kazakhstan/NLA/barcode47-10-MN908947.3/2022 | EPI_ISL_13717829 | 2022-01-09 | 30 | Female | 19.07 | BA.1.1 | 0.36 | 269X | 1283 |
| 160 | hCoV-19/Kazakhstan/NLA/barcode48-10-MN908947.3/2022 | EPI_ISL_13717830 | 2022-01-09 | 35 | Female | 13.42 | BA.1.1 | 0.36 | 280X | 1256 |
| 161 | hCoV-19/Kazakhstan/NLA/barcode49-10-MN908947.3/2022 | EPI_ISL_13717831 | 2022-01-05 | 59 | Female | 17.24 | BA.1.1 | 0.36 | 274X | 1502 |
| 162 | hCoV-19/Kazakhstan/NLA/barcode52-10-MN908947.3/2022 | EPI_ISL_13717832 | 2022-01-05 | 23 | Female | 16.99 | BA.1.1 | 0.35 | 237X | 2637 |
| 163 | hCoV-19/Kazakhstan/NLA/barcode57-10-MN908947.3/2022 | EPI_ISL_13717833 | 2022-01-05 | 29 | Female | 15.95 | BA.1.1 | 0.35 | 243X | 2712 |
| 164 | hCoV-19/Kazakhstan/NLA/barcode58-10-MN908947.3/2022 | EPI_ISL_13717834 | 2022-01-05 | 35 | Female | 14.82 | BA.1.1 | 0.36 | 214X | 1538 |
| 165 | hCoV-19/Kazakhstan/NLA/barcode65-10-MN908947.3/2022 | EPI_ISL_13717835 | 2022-01-06 | 35 | Male | 20.82 | BA.1.1 | 0.36 | 198X | 1711 |
| 166 | hCoV-19/Kazakhstan/NLA/barcode67-10-MN908947.3/2022 | EPI_ISL_13717836 | 2022-01-06 | 38 | Male | 18.05 | BA.1.1 | 0.35 | 273X | 2362 |
| 167 | hCoV-19/Kazakhstan/NLA/barcode68-10-MN908947.3/2022 | EPI_ISL_13717837 | 2022-01-06 | 18 | Female | 13.69 | BA.1.1 | 0.36 | 307X | 1480 |
| 168 | hCoV-19/Kazakhstan/NLA/barcode69-10-MN908947.3/2022 | EPI_ISL_13717838 | 2022-01-06 | 12 | Female | 18.98 | BA.1.1 | 0.36 | 233X | 1768 |
| 169 | hCoV-19/Kazakhstan/NLA/barcode70-10-MN908947.3/2022 | EPI_ISL_13717839 | 2022-01-06 | 10 | Female | 19.45 | BA.1.1 | 0.34 | 179X | 2879 |
| 170 | hCoV-19/Kazakhstan/NLA/barcode73-10-MN908947.3/2022 | EPI_ISL_13717840 | 2022-01-06 | 26 | Male | 18.1 | AY.122 | 0.37 | 252X | 863 |
| 171 | hCoV-19/Kazakhstan/NLA/barcode75-10-MN908947.3/2022 | EPI_ISL_13717841 | 2022-01-06 | 38 | Female | 13.51 | BA.1.1 | 0.36 | 258X | 1486 |
| 172 | hCoV-19/Kazakhstan/NLA/barcode77-10-MN908947.3/2022 | EPI_ISL_13717842 | 2022-01-06 | 45 | Female | 19.95 | BA.1.1 | 0.36 | 203X | 1739 |
| 173 | hCoV-19/Kazakhstan/NLA/barcode80-10-MN908947.3/2022 | EPI_ISL_13717843 | 2022-01-08 | 38 | Male | 15.36 | BA.1 | 0.35 | 206X | 2038 |
| 174 | hCoV-19/Kazakhstan/NLA/barcode82-10-MN908947.3/2022 | EPI_ISL_13717844 | 2022-01-08 | 57 | Female | 21.53 | BA.1.1 | 0.36 | 240X | 1509 |
| 175 | hCoV-19/Kazakhstan/NLA/barcode85-10-MN908947.3/2022 | EPI_ISL_13717845 | 2022-01-08 | 46 | Female | 16.43 | BA.1.1 | 0.36 | 265X | 1524 |
| 176 | hCoV-19/Kazakhstan/NLA/barcode87-10-MN908947.3/2022 | EPI_ISL_13717846 | 2022-01-08 | 42 | Female | 17.07 | BA.1.1 | 0.36 | 242X | 1503 |
| 177 | hCoV-19/Kazakhstan/NLA/barcode88-10-MN908947.3/2022 | EPI_ISL_13717847 | 2022-01-08 | 34 | Female | 17.76 | BA.1.1 | 0.35 | 206X | 2475 |
| 178 | hCoV-19/Kazakhstan/NLA/barcode89-10-MN908947.3/2022 | EPI_ISL_13717848 | 2022-01-08 | 24 | Female | 21.36 | BA.1.1 | 0.36 | 271X | 1480 |
| 179 | hCoV-19/Kazakhstan/NLA/barcode90-10-MN908947.3/2022 | EPI_ISL_13717849 | 2022-01-08 | 61 | Female | 19.55 | BA.1.1 | 0.36 | 226X | 1477 |
| 180 | hCoV-19/Kazakhstan/NLA/barcode91-10-MN908947.3/2022 | EPI_ISL_13717850 | 2022-01-08 | 28 | Female | 17.25 | BA.1 | 0.36 | 253X | 1512 |
| 181 | hCoV-19/Kazakhstan/NLA/barcode04-11-MN908947.3/2022 | EPI_ISL_13717851 | 2022-01-11 | 42 | Female | 12.47 | BA.1 | 0.35 | 238X | 2340 |
| 182 | hCoV-19/Kazakhstan/NLA/barcode08-11-MN908947.3/2022 | EPI_ISL_13717852 | 2022-01-11 | 56 | Female | 14.48 | BA.1.1 | 0.35 | 252X | 2198 |
| 183 | hCoV-19/Kazakhstan/NLA/barcode60-11-MN908947.3/2022 | EPI_ISL_13717853 | 2022-01-12 | 19 | Female | 12.75 | BA.1.1 | 0.37 | 256X | 523 |
| 184 | hCoV-19/Kazakhstan/NLA/barcode64-11-MN908947.3/2022 | EPI_ISL_13717854 | 2022-01-12 | 22 | Male | 17.01 | BA.1.1 | 0.35 | 257X | 2417 |
| 185 | hCoV-19/Kazakhstan/NLA/barcode19-12-MN908947.3/2022 | EPI_ISL_13717855 | 2022-01-10 | 22 | Male | 16.99 | BA.1.1 | 0.36 | 221X | 1874 |
| 186 | hCoV-19/Kazakhstan/NLA/barcode25-12-MN908947.3/2022 | EPI_ISL_13717856 | 2022-01-10 | 47 | Male | 14.6 | BA.1.1 | 0.36 | 184X | 1815 |
| 187 | hCoV-19/Kazakhstan/NLA/barcode42-13-MN908947.3/2022 | EPI_ISL_13717857 | 2022-01-22 | 21 | Female | 15.14 | BA.1.1 | 0.36 | 263X | 1039 |
| 188 | hCoV-19/Kazakhstan/NLA/barcode43-13-MN908947.3/2022 | EPI_ISL_13717858 | 2022-01-22 | 72 | Male | 17.41 | BA.1.1 | 0.36 | 228X | 1466 |
| 189 | hCoV-19/Kazakhstan/NLA/barcode47-13-MN908947.3/2022 | EPI_ISL_13717859 | 2022-01-22 | 56 | Male | 17.49 | BA.1.1 | 0.36 | 257X | 1122 |
| 190 | hCoV-19/Kazakhstan/NLA/barcode81-13-MN908947.3/2022 | EPI_ISL_13717860 | 2022-01-24 | 1 | Male | 13.58 | BA.1.1 | 0.36 | 88X | 1362 |
| 191 | hCoV-19/Kazakhstan/NLA/barcode03-14-MN908947.3/2022 | EPI_ISL_13717861 | 2022-01-14 | 31 | Female | 15.15 | BA.1.1 | 0.35 | 262X | 2373 |
| 192 | hCoV-19/Kazakhstan/NLA/barcode04-14-MN908947.3/2022 | EPI_ISL_13717862 | 2022-01-14 | 9 | Male | 17.44 | BA.1.1 | 0.34 | 245X | 2987 |
| 193 | hCoV-19/Kazakhstan/NLA/barcode20-14-MN908947.3/2022 | EPI_ISL_13717863 | 2022-01-15 | 46 | Male | 17.9 | BA.1.1 | 0.35 | 236X | 2511 |
| 194 | hCoV-19/Kazakhstan/NLA/barcode39-14-MN908947.3/2022 | EPI_ISL_13717864 | 2022-01-17 | 45 | Female | 16.6 | BA.1.1 | 0.36 | 270X | 1332 |
| 195 | hCoV-19/Kazakhstan/NLA/barcode41-14-MN908947.3/2022 | EPI_ISL_13717865 | 2022-01-17 | 22 | Male | 13.88 | BA.1.1 | 0.37 | 302X | 1083 |
| 196 | hCoV-19/Kazakhstan/NLA/barcode48-14-MN908947.3/2022 | EPI_ISL_13717866 | 2022-01-17 | 59 | Female | 14.68 | BA.1 | 0.36 | 243X | 1725 |
| 197 | hCoV-19/Kazakhstan/NLA/barcode01-15-MN908947.3/2022 | EPI_ISL_13717867 | 2022-01-31 | 20 | Female | 21.82 | BA.1.1 | 0.36 | 145X | 1332 |
| 198 | hCoV-19/Kazakhstan/NLA/barcode02-15-MN908947.3/2022 | EPI_ISL_13717868 | 2022-01-31 | 23 | Male | 19.31 | BA.1.1 | 0.36 | 189X | 1463 |
| 199 | hCoV-19/Kazakhstan/NLA/barcode03-15-MN908947.3/2022 | EPI_ISL_13717869 | 2022-01-31 | 20 | Female | 20.81 | BA.1.1 | 0.36 | 197X | 1352 |
| 200 | hCoV-19/Kazakhstan/NLA/barcode04-15-MN908947.3/2022 | EPI_ISL_13717870 | 2022-01-31 | 22 | Female | 18.41 | BA.1.1 | 0.36 | 213X | 1283 |
| 201 | hCoV-19/Kazakhstan/NLA/barcode05-15-MN908947.3/2022 | EPI_ISL_13717871 | 2022-02-01 | 32 | Male | 18.84 | BA.1.1 | 0.36 | 122X | 1795 |
| 202 | hCoV-19/Kazakhstan/NLA/barcode06-15-MN908947.3/2022 | EPI_ISL_13717872 | 2022-02-01 | 7 | Female | 13.79 | BA.1.1 | 0.36 | 192X | 1301 |
| 203 | hCoV-19/Kazakhstan/NLA/barcode07-15-MN908947.3/2022 | EPI_ISL_13717873 | 2022-02-01 | 35 | Female | 18.81 | BA.1.1 | 0.35 | 171X | 2380 |
| 204 | hCoV-19/Kazakhstan/NLA/barcode08-15-MN908947.3/2022 | EPI_ISL_13717874 | 2022-02-01 | 45 | Female | 13.34 | BA.1.1 | 0.36 | 171X | 1875 |
| 205 | hCoV-19/Kazakhstan/NLA/barcode09-15-MN908947.3/2022 | EPI_ISL_13717875 | 2022-02-01 | 26 | Female | 16.85 | BA.1.1 | 0.36 | 227X | 1333 |
| 206 | hCoV-19/Kazakhstan/NLA/barcode10-15-MN908947.3/2022 | EPI_ISL_13717876 | 2022-02-01 | 51 | Male | 21.72 | BA.1.1 | 0.34 | 61X | 2687 |
| 207 | hCoV-19/Kazakhstan/NLA/barcode11-15-MN908947.3/2022 | EPI_ISL_13717877 | 2022-02-02 | 41 | Male | 20.4 | BA.1.1 | 0.36 | 180X | 1323 |
| 208 | hCoV-19/Kazakhstan/NLA/barcode12-15-MN908947.3/2022 | EPI_ISL_13717878 | 2022-02-02 | 77 | Male | 18.91 | BA.1.1 | 0.36 | 116X | 1516 |
| 209 | hCoV-19/Kazakhstan/NLA/barcode14-15-MN908947.3/2022 | EPI_ISL_13717879 | 2022-02-03 | 57 | Male | 15.54 | BA.1.1 | 0.36 | 198X | 1385 |
| 210 | hCoV-19/Kazakhstan/NLA/barcode15-15-MN908947.3/2022 | EPI_ISL_13717880 | 2022-02-03 | 23 | Female | 18.86 | BA.1.1 | 0.36 | 195X | 1345 |
| 211 | hCoV-19/Kazakhstan/NLA/barcode16-15-MN908947.3/2022 | EPI_ISL_13717881 | 2022-02-03 | 21 | Male | 17.19 | BA.1.1 | 0.36 | 173X | 1375 |
| 212 | hCoV-19/Kazakhstan/NLA/barcode17-15-MN908947.3/2022 | EPI_ISL_13717882 | 2022-02-04 | 32 | Female | 21.52 | BA.1.1 | 0.35 | 152X | 2150 |
| 213 | hCoV-19/Kazakhstan/NLA/barcode18-15-MN908947.3/2022 | EPI_ISL_13717883 | 2022-02-04 | 23 | Male | 21.16 | BA.1.1 | 0.36 | 141X | 1434 |
| 214 | hCoV-19/Kazakhstan/NLA/barcode19-15-MN908947.3/2022 | EPI_ISL_13717884 | 2022-02-04 | 8 | Male | 17.28 | BA.1.1 | 0.36 | 202X | 1350 |
| 215 | hCoV-19/Kazakhstan/NLA/barcode20-15-MN908947.3/2022 | EPI_ISL_13717885 | 2022-02-04 | 4 | Male | 19.11 | BA.1.1 | 0.36 | 114X | 1544 |
| 216 | hCoV-19/Kazakhstan/NLA/barcode21-15-MN908947.3/2022 | EPI_ISL_13717886 | 2022-02-04 | 23 | Female | 16.54 | BA.1.1 | 0.36 | 163X | 1357 |
| 217 | hCoV-19/Kazakhstan/NLA/barcode22-15-MN908947.3/2022 | EPI_ISL_13717887 | 2022-02-04 | 20 | Female | 21.2 | BA.1.1 | 0.36 | 143X | 1426 |
| 218 | hCoV-19/Kazakhstan/NLA/barcode23-15-MN908947.3/2022 | EPI_ISL_13717888 | 2022-02-05 | 67 | Female | 20.51 | BA.1.1 | 0.36 | 123X | 1345 |
| 219 | hCoV-19/Kazakhstan/NLA/barcode24-15-MN908947.3/2022 | EPI_ISL_13717889 | 2022-02-05 | 45 | Female | 19.44 | BA.1.1 | 0.36 | 190X | 1332 |
| 220 | hCoV-19/Kazakhstan/NLA/barcode25-15-MN908947.3/2022 | EPI_ISL_13717890 | 2022-02-05 | 68 | Female | 14.32 | BA.1.1 | 0.35 | 227X | 2355 |
| 221 | hCoV-19/Kazakhstan/NLA/barcode26-15-MN908947.3/2022 | EPI_ISL_13717891 | 2022-02-07 | 21 | Female | 15.08 | BA.1.1 | 0.36 | 236X | 1331 |
| 222 | hCoV-19/Kazakhstan/NLA/barcode27-15-MN908947.3/2022 | EPI_ISL_13717892 | 2022-02-07 | 41 | Female | 19.54 | BA.1.1 | 0.36 | 153X | 1397 |
| 223 | hCoV-19/Kazakhstan/NLA/barcode28-15-MN908947.3/2022 | EPI_ISL_13717893 | 2022-02-07 | 49 | Male | 13.07 | BA.1.1 | 0.36 | 249X | 1321 |
| 224 | hCoV-19/Kazakhstan/NLA/barcode29-15-MN908947.3/2022 | EPI_ISL_13717894 | 2022-02-07 | 7 | Female | 17.29 | BA.1.1 | 0.35 | 136X | 1962 |
| 225 | hCoV-19/Kazakhstan/NLA/barcode30-15-MN908947.3/2022 | EPI_ISL_13717895 | 2022-02-08 | 21 | Male | 22.13 | BA.1.1 | 0.35 | 104X | 2557 |
| 226 | hCoV-19/Kazakhstan/NLA/barcode31-15-MN908947.3/2022 | EPI_ISL_13717896 | 2022-02-09 | 32 | Female | 17.65 | BA.1.1 | 0.36 | 179X | 1383 |
| 227 | hCoV-19/Kazakhstan/NLA/barcode33-15-MN908947.3/2022 | EPI_ISL_13717897 | 2022-02-09 | 55 | Male | 14.91 | BA.1.1 | 0.36 | 173X | 1326 |
| 228 | hCoV-19/Kazakhstan/NLA/barcode34-15-MN908947.3/2022 | EPI_ISL_13717898 | 2022-02-09 | 33 | Female | 15.66 | BA.1.1 | 0.36 | 204X | 1321 |
| 229 | hCoV-19/Kazakhstan/NLA/barcode35-15-MN908947.3/2022 | EPI_ISL_13717899 | 2022-02-09 | 21 | Female | 22.7 | BA.1 | 0.37 | 96X | 945 |
| 230 | hCoV-19/Kazakhstan/NLA/barcode36-15-MN908947.3/2022 | EPI_ISL_13717900 | 2022-02-10 | 35 | Male | 20.15 | BA.1.1 | 0.36 | 142X | 1408 |
| 231 | hCoV-19/Kazakhstan/NLA/barcode37-15-MN908947.3/2022 | EPI_ISL_13717901 | 2022-02-10 | unknown | unknown | unknown | BA.1.1 | 0.36 | 186X | 1362 |
| 232 | hCoV-19/Kazakhstan/NLA/barcode39-15-MN908947.3/2022 | EPI_ISL_13717902 | 2022-02-11 | 22 | Female | 12.97 | BA.1.1 | 0.36 | 229X | 1377 |
| 233 | hCoV-19/Kazakhstan/NLA/barcode40-15-MN908947.3/2022 | EPI_ISL_13717903 | 2022-02-12 | 19 | Male | 18.29 | BA.1.1 | 0.35 | 228X | 2033 |
| 234 | hCoV-19/Kazakhstan/NLA/barcode41-15-MN908947.3/2022 | EPI_ISL_13717904 | 2022-02-15 | 21 | Female | 19.64 | BA.1.1 | 0.35 | 171X | 2345 |
| 235 | hCoV-19/Kazakhstan/NLA/barcode42-15-MN908947.3/2022 | EPI_ISL_13717905 | 2022-02-15 | 22 | Male | 18.71 | BA.1.1 | 0.36 | 97X | 1436 |
| 236 | hCoV-19/Kazakhstan/NLA/barcode43-15-MN908947.3/2022 | EPI_ISL_13717906 | 2022-02-16 | 38 | Female | 16.26 | BA.1.15 | 0.36 | 205X | 1397 |
| 237 | hCoV-19/Kazakhstan/NLA/barcode44-15-MN908947.3/2022 | EPI_ISL_13717907 | 2022-02-16 | 42 | Female | 17.08 | BA.1.1 | 0.35 | 230X | 2345 |
| 238 | hCoV-19/Kazakhstan/NLA/barcode45-15-MN908947.3/2022 | EPI_ISL_13717908 | 2022-02-17 | 20 | Male | 15.25 | BA.1.1 | 0.36 | 251X | 1318 |
| 239 | hCoV-19/Kazakhstan/NLA/barcode46-15-MN908947.3/2022 | EPI_ISL_13717909 | 2022-02-21 | 39 | Male | 18.03 | BA.1.1 | 0.36 | 153X | 1408 |
| 240 | hCoV-19/Kazakhstan/NLA/barcode47-15-MN908947.3/2022 | EPI_ISL_13717910 | 2022-02-21 | 43 | Male | 20.66 | BA.1.1 | 0.35 | 140X | 2391 |
| 241 | hCoV-19/Kazakhstan/NLA/barcode48-15-MN908947.3/2022 | EPI_ISL_13717911 | 2022-02-22 | 23 | Male | 21.37 | BA.1 | 0.36 | 102X | 1271 |
| 242 | hCoV-19/Kazakhstan/NLA/barcode49-15-MN908947.3/2022 | EPI_ISL_13717912 | 2022-03-01 | 53 | Female | 19.25 | BA.1.1 | 0.36 | 208X | 1334 |
| 243 | hCoV-19/Kazakhstan/NLA/barcode50-15-MN908947.3/2022 | EPI_ISL_13717913 | 2022-03-04 | 23 | Female | 13.32 | BA.2 | 0.35 | 213X | 1800 |
| 244 | hCoV-19/Kazakhstan/NLA/barcode53-15-MN908947.3/2022 | EPI_ISL_13717914 | 2022-03-10 | 33 | Male | 17.5 | BA.2 | 0.36 | 218X | 1759 |
| 245 | hCoV-19/Kazakhstan/NLA/barcode56-15-MN908947.3/2022 | EPI_ISL_13717915 | 2022-05-28 | 36 | Female | 16.11 | BA.2 | 0.36 | 232X | 1660 |
| 246 | hCoV-19/Kazakhstan/NLA/barcode58-15-MN908947.3/2022 | EPI_ISL_13717916 | 2022-01-13 | 22 | Female | 18.16 | BA.1.1 | 0.36 | 115X | 1418 |
| 247 | hCoV-19/Kazakhstan/NLA/barcode59-15-MN908947.3/2022 | EPI_ISL_13717917 | 2022-01-13 | 42 | Female | 17.36 | BA.1.1 | 0.36 | 194X | 1360 |
| 248 | hCoV-19/Kazakhstan/NLA/barcode61-15-MN908947.3/2022 | EPI_ISL_13717918 | 2022-01-13 | 44 | Female | 19.82 | BA.1.1 | 0.36 | 167X | 1359 |
| 249 | hCoV-19/Kazakhstan/NLA/barcode62-15-MN908947.3/2022 | EPI_ISL_13717919 | 2022-01-19 | 30 | Female | 19.55 | BA.1.1 | 0.36 | 115X | 1507 |
| 250 | hCoV-19/Kazakhstan/NLA/barcode63-15-MN908947.3/2022 | EPI_ISL_13717920 | 2022-01-19 | 25 | Male | 19.27 | BA.1 | 0.37 | 173X | 526 |
| 251 | hCoV-19/Kazakhstan/NLA/barcode64-15-MN908947.3/2022 | EPI_ISL_13717921 | 2022-01-19 | 7 | Male | 17.18 | BA.1.1 | 0.36 | 229X | 1333 |
| 252 | hCoV-19/Kazakhstan/NLA/barcode65-15-MN908947.3/2022 | EPI_ISL_13717922 | 2022-01-19 | 31 | Male | 20.93 | BA.1.1 | 0.36 | 152X | 1395 |
| 253 | hCoV-19/Kazakhstan/NLA/barcode66-15-MN908947.3/2022 | EPI_ISL_13717923 | 2022-01-19 | 28 | Female | 20.29 | BA.1.1 | 0.35 | 138X | 2473 |
| 254 | hCoV-19/Kazakhstan/NLA/barcode67-15-MN908947.3/2022 | EPI_ISL_13717924 | 2022-01-19 | 19 | Female | 17.6 | BA.1.1 | 0.36 | 233X | 1332 |
| 255 | hCoV-19/Kazakhstan/NLA/barcode68-15-MN908947.3/2022 | EPI_ISL_13717925 | 2022-01-19 | 41 | Female | 18.22 | BA.1.1 | 0.36 | 196X | 1338 |
| 256 | hCoV-19/Kazakhstan/NLA/barcode69-15-MN908947.3/2022 | EPI_ISL_13717926 | 2022-01-19 | 34 | Female | 17.53 | BA.1.1 | 0.36 | 200X | 1341 |
| 257 | hCoV-19/Kazakhstan/NLA/barcode70-15-MN908947.3/2022 | EPI_ISL_13717927 | 2022-01-20 | 42 | Female | 15.77 | BA.1.1 | 0.36 | 199X | 1323 |
| 258 | hCoV-19/Kazakhstan/NLA/barcode71-15-MN908947.3/2022 | EPI_ISL_13717928 | 2022-01-20 | 49 | Female | 20.31 | BA.1.1 | 0.36 | 157X | 1379 |
| 259 | hCoV-19/Kazakhstan/NLA/barcode72-15-MN908947.3/2022 | EPI_ISL_13717929 | 2022-01-20 | 42 | Female | 17.2 | BA.1.1 | 0.36 | 180X | 1474 |
| 260 | hCoV-19/Kazakhstan/NLA/barcode73-15-MN908947.3/2022 | EPI_ISL_13717930 | 2022-01-20 | 28 | Male | 18.17 | BA.1.1 | 0.35 | 200X | 1925 |
| 261 | hCoV-19/Kazakhstan/NLA/barcode74-15-MN908947.3/2022 | EPI_ISL_13717931 | 2022-01-20 | 18 | Female | 15.95 | BA.1.1 | 0.36 | 204X | 1420 |
| 262 | hCoV-19/Kazakhstan/NLA/barcode76-15-MN908947.3/2022 | EPI_ISL_13717932 | 2022-01-20 | 28 | Male | 20.27 | BA.1.1 | 0.35 | 130X | 2165 |
| 263 | hCoV-19/Kazakhstan/NLA/barcode77-15-MN908947.3/2022 | EPI_ISL_13717933 | 2022-01-20 | 18 | Female | 14.64 | BA.1.1 | 0.35 | 119X | 2316 |
| 264 | hCoV-19/Kazakhstan/NLA/barcode78-15-MN908947.3/2022 | EPI_ISL_13717934 | 2022-01-20 | 34 | Male | 18.06 | BA.1.1 | 0.35 | 150X | 2351 |
| 265 | hCoV-19/Kazakhstan/NLA/barcode81-15-MN908947.3/2022 | EPI_ISL_13717935 | 2022-01-21 | 43 | Male | 17.19 | BA.1.1 | 0.35 | 164X | 2286 |
| 266 | hCoV-19/Kazakhstan/NLA/barcode83-15-MN908947.3/2022 | EPI_ISL_13717936 | 2022-01-21 | 21 | Male | 18.22 | BA.1 | 0.36 | 155X | 1392 |
| 267 | hCoV-19/Kazakhstan/NLA/barcode84-15-MN908947.3/2022 | EPI_ISL_13717937 | 2022-01-21 | 62 | Male | 15.83 | BA.1.1 | 0.36 | 191X | 1345 |
| 268 | hCoV-19/Kazakhstan/NLA/barcode85-15-MN908947.3/2022 | EPI_ISL_13717938 | 2022-01-13 | 73 | Female | 19.18 | BA.1.1 | 0.35 | 101X | 2591 |
| 269 | hCoV-19/Kazakhstan/NLA/barcode89-15-MN908947.3/2022 | EPI_ISL_13717939 | 2022-01-14 | 53 | Male | 24.98 | BA.1.1 | 0.35 | 88X | 2554 |
| 270 | hCoV-19/Kazakhstan/NLA/barcode91-15-MN908947.3/2022 | EPI_ISL_13717940 | 2022-01-14 | 28 | Female | 23.79 | BA.1 | 0.36 | 101X | 1558 |
| 271 | hCoV-19/Kazakhstan/NLA/barcode92-15-MN908947.3/2022 | EPI_ISL_13717941 | 2022-01-13 | 47 | Male | 20.58 | BA.1.1 | 0.35 | 128X | 2520 |
| 272 | hCoV-19/Kazakhstan/NLA/barcode74-16-MN908947.3/2022 | EPI_ISL_13717942 | 2022-01-26 | 25 | Male | 11.28 | BA.1.1 | 0.36 | 273X | 1972 |
| 273 | hCoV-19/Kazakhstan/NLA/barcode86-16-MN908947.3/2022 | EPI_ISL_13717943 | 2022-01-28 | 32 | Female | 14.14 | BA.1.1 | 0.35 | 222X | 2518 |
| 274 | hCoV-19/Kazakhstan/NLA/barcode20-17-MN908947.3/2022 | EPI_ISL_13717944 | 2022-01-14 | 24 | Female | 21.99 | BA.1.1 | 0.34 | 92X | 2934 |
| 275 | hCoV-19/Kazakhstan/NLA/barcode54-17-MN908947.3/2022 | EPI_ISL_13717945 | 2022-01-17 | 25 | Male | 22.14 | BA.1.1 | 0.34 | 61X | 2990 |
| 276 | hCoV-19/Kazakhstan/NLA/barcode67-17-MN908947.3/2022 | EPI_ISL_13717946 | 2022-01-17 | 44 | Female | 20.28 | BA.1.1 | 0.35 | 113X | 2397 |
| 277 | hCoV-19/Kazakhstan/NLA/barcode78-17-MN908947.3/2022 | EPI_ISL_13717947 | 2022-01-24 | 29 | Female | 14.17 | BA.1.1 | 0.35 | 114X | 2474 |
| 278 | hCoV-19/Kazakhstan/NLA/barcode85-17-MN908947.3/2022 | EPI_ISL_13717948 | 2022-01-09 | 43 | Female | 20.63 | BA.1.1 | 0.35 | 64X | 2575 |
| 279 | hCoV-19/Kazakhstan/NLA/barcode89-17-MN908947.3/2022 | EPI_ISL_13717949 | 2022-01-10 | 50 | Female | 18.67 | BA.1.1 | 0.35 | 109X | 2140 |
| 280 | hCoV-19/Kazakhstan/NLA/barcode93-17-MN908947.3/2022 | EPI_ISL_13717950 | 2022-01-11 | 41 | Male | 19.68 | BA.1.1 | 0.34 | 103X | 2844 |
| 281 | hCoV-19/Kazakhstan/NLA/barcode94-17-MN908947.3/2022 | EPI_ISL_13717951 | 2022-01-11 | 60 | Female | 21.65 | BA.1.1 | 0.35 | 89X | 2485 |
| 282 | hCoV-19/Kazakhstan/NLA/barcode95-17-MN908947.3/2022 | EPI_ISL_13717952 | 2022-01-11 | 40 | Male | 19.89 | BA.1.1 | 0.34 | 98X | 2910 |
| 283 | hCoV-19/Kazakhstan/NLA/barcode01-18-MN908947.3/2022 | EPI_ISL_13717953 | 2022-01-11 | 30 | Female | 18.16 | BA.1.1 | 0.36 | 162X | 1355 |
| 284 | hCoV-19/Kazakhstan/NLA/barcode02-18-MN908947.3/2022 | EPI_ISL_13717954 | 2022-01-11 | 33 | Female | 19.63 | BA.1.1 | 0.36 | 166X | 1407 |
| 285 | hCoV-19/Kazakhstan/NLA/barcode03-18-MN908947.3/2022 | EPI_ISL_13717955 | 2022-01-11 | 23 | Male | 15.57 | BA.1.1 | 0.36 | 261X | 1283 |
| 286 | hCoV-19/Kazakhstan/NLA/barcode04-18-MN908947.3/2022 | EPI_ISL_13717956 | 2022-01-11 | 27 | Female | 21.3 | BA.1.1 | 0.36 | 189X | 1646 |
| 287 | hCoV-19/Kazakhstan/NLA/barcode05-18-MN908947.3/2022 | EPI_ISL_13717957 | 2022-01-11 | 60 | Female | 18.41 | BA.1.1 | 0.35 | 117X | 2428 |
| 288 | hCoV-19/Kazakhstan/NLA/barcode09-18-MN908947.3/2022 | EPI_ISL_13717958 | 2022-01-11 | 57 | Female | 22.36 | BA.1.1 | 0.35 | 115X | 2260 |
| 289 | hCoV-19/Kazakhstan/NLA/barcode11-18-MN908947.3/2022 | EPI_ISL_13717959 | 2022-01-12 | 26 | Male | 24.03 | BA.1.1 | 0.36 | 145X | 1486 |
| 290 | hCoV-19/Kazakhstan/NLA/barcode12-18-MN908947.3/2022 | EPI_ISL_13717960 | 2022-01-12 | 20 | Male | 15.62 | BA.1.1 | 0.36 | 199X | 1352 |
| 291 | hCoV-19/Kazakhstan/NLA/barcode13-18-MN908947.3/2022 | EPI_ISL_13717961 | 2022-01-12 | 42 | Male | 18.34 | BA.1.1 | 0.36 | 222X | 1284 |
| 292 | hCoV-19/Kazakhstan/NLA/barcode14-18-MN908947.3/2022 | EPI_ISL_13717962 | 2022-01-12 | 35 | Female | 21.39 | BA.1.1 | 0.36 | 140X | 1395 |
| 293 | hCoV-19/Kazakhstan/NLA/barcode17-18-MN908947.3/2022 | EPI_ISL_13717963 | 2022-01-12 | 30 | Male | 20.01 | BA.1.1 | 0.36 | 208X | 1291 |
| 294 | hCoV-19/Kazakhstan/NLA/barcode18-18-MN908947.3/2022 | EPI_ISL_13717964 | 2022-01-12 | 41 | Male | 22.07 | BA.1.1 | 0.36 | 137X | 1436 |
| 295 | hCoV-19/Kazakhstan/NLA/barcode19-18-MN908947.3/2022 | EPI_ISL_13717965 | 2022-01-12 | 21 | Female | 21.01 | BA.1.1 | 0.36 | 136X | 1501 |
| 296 | hCoV-19/Kazakhstan/NLA/barcode20-18-MN908947.3/2022 | EPI_ISL_13717966 | 2022-01-12 | 22 | Female | 20.48 | BA.1.1 | 0.35 | 95X | 2338 |
| 297 | hCoV-19/Kazakhstan/NLA/barcode21-18-MN908947.3/2022 | EPI_ISL_13717967 | 2022-01-12 | 24 | Female | 21.99 | BA.1.1 | 0.36 | 160X | 1814 |
| 298 | hCoV-19/Kazakhstan/NLA/barcode22-18-MN908947.3/2022 | EPI_ISL_13717968 | 2022-01-12 | 61 | Female | 23.23 | BA.1.1 | 0.36 | 169X | 1333 |
| 299 | hCoV-19/Kazakhstan/NLA/barcode23-18-MN908947.3/2022 | EPI_ISL_13717969 | 2022-01-12 | 38 | Male | 13.97 | BA.1.1 | 0.36 | 179X | 1444 |
| 300 | hCoV-19/Kazakhstan/NLA/barcode24-18-MN908947.3/2022 | EPI_ISL_13717970 | 2022-01-12 | 47 | Female | 16.86 | BA.1.1 | 0.36 | 258X | 1287 |
| 301 | hCoV-19/Kazakhstan/NLA/barcode25-18-MN908947.3/2022 | EPI_ISL_13717971 | 2022-01-13 | 51 | Female | 19.29 | BA.1.1 | 0.36 | 233X | 1304 |
| 302 | hCoV-19/Kazakhstan/NLA/barcode26-18-MN908947.3/2022 | EPI_ISL_13717972 | 2022-01-13 | 29 | Male | 20.68 | BA.1.1 | 0.36 | 219X | 1302 |
| 303 | hCoV-19/Kazakhstan/NLA/barcode27-18-MN908947.3/2022 | EPI_ISL_13717973 | 2022-01-13 | 46 | Female | 12.81 | BA.1.1 | 0.36 | 252X | 1330 |
| 304 | hCoV-19/Kazakhstan/NLA/barcode28-18-MN908947.3/2022 | EPI_ISL_13717974 | 2022-01-13 | 30 | Male | 17.51 | BA.1.1 | 0.36 | 250X | 1283 |
| 305 | hCoV-19/Kazakhstan/NLA/barcode30-18-MN908947.3/2022 | EPI_ISL_13717975 | 2022-01-13 | 31 | Male | 18.82 | BA.1 | 0.37 | 246X | 377 |
| 306 | hCoV-19/Kazakhstan/NLA/barcode31-18-MN908947.3/2022 | EPI_ISL_13717976 | 2022-01-13 | 30 | Female | 21.72 | BA.1 | 0.36 | 128X | 1560 |
| 307 | hCoV-19/Kazakhstan/NLA/barcode32-18-MN908947.3/2022 | EPI_ISL_13717977 | 2022-01-13 | 45 | Male | 18.91 | BA.1.1 | 0.35 | 142X | 2097 |
| 308 | hCoV-19/Kazakhstan/NLA/barcode33-18-MN908947.3/2022 | EPI_ISL_13717978 | 2022-01-13 | 33 | Female | 21.03 | BA.1.1 | 0.36 | 122X | 1509 |
| 309 | hCoV-19/Kazakhstan/NLA/barcode34-18-MN908947.3/2022 | EPI_ISL_13717979 | 2022-01-13 | 71 | Female | 16.59 | BA.1.1 | 0.36 | 233X | 1317 |
| 310 | hCoV-19/Kazakhstan/NLA/barcode36-18-MN908947.3/2022 | EPI_ISL_13717980 | 2022-01-13 | 41 | Female | 19.24 | BA.1.1 | 0.36 | 187X | 1645 |
| 311 | hCoV-19/Kazakhstan/NLA/barcode37-18-MN908947.3/2022 | EPI_ISL_13717981 | 2022-01-13 | 24 | Male | 18.16 | BA.1.1 | 0.36 | 238X | 1688 |
| 312 | hCoV-19/Kazakhstan/NLA/barcode39-18-MN908947.3/2022 | EPI_ISL_13717982 | 2022-01-21 | 48 | Female | 22.98 | BA.1.1 | 0.35 | 107X | 2474 |
| 313 | hCoV-19/Kazakhstan/NLA/barcode40-18-MN908947.3/2022 | EPI_ISL_13717983 | 2022-01-21 | 28 | Female | 19.56 | BA.1.1 | 0.36 | 216X | 1316 |
| 314 | hCoV-19/Kazakhstan/NLA/barcode41-18-MN908947.3/2022 | EPI_ISL_13717984 | 2022-01-22 | 39 | Male | 17.01 | BA.1.1 | 0.36 | 276X | 1284 |
| 315 | hCoV-19/Kazakhstan/NLA/barcode43-18-MN908947.3/2022 | EPI_ISL_13717985 | 2022-01-14 | 46 | Female | 18.26 | BA.1.1 | 0.36 | 124X | 1434 |
| 316 | hCoV-19/Kazakhstan/NLA/barcode44-18-MN908947.3/2022 | EPI_ISL_13717986 | 2022-01-14 | 34 | Female | 18.74 | BA.1.1 | 0.36 | 167X | 1826 |
| 317 | hCoV-19/Kazakhstan/NLA/barcode45-18-MN908947.3/2022 | EPI_ISL_13717987 | 2022-01-14 | 31 | Female | 17.55 | BA.1.1 | 0.36 | 169X | 1376 |
| 318 | hCoV-19/Kazakhstan/NLA/barcode46-18-MN908947.3/2022 | EPI_ISL_13717988 | 2022-01-14 | 30 | Female | 16.51 | BA.1.1 | 0.36 | 263X | 1274 |
| 319 | hCoV-19/Kazakhstan/NLA/barcode48-18-MN908947.3/2022 | EPI_ISL_13717989 | 2022-01-15 | 40 | Male | 14.21 | BA.1.1 | 0.36 | 210X | 1364 |
| 320 | hCoV-19/Kazakhstan/NLA/barcode49-18-MN908947.3/2022 | EPI_ISL_13717990 | 2022-01-15 | 29 | Female | 18.78 | BA.1.1 | 0.36 | 118X | 1709 |
| 321 | hCoV-19/Kazakhstan/NLA/barcode50-18-MN908947.3/2022 | EPI_ISL_13717991 | 2022-01-15 | 46 | Male | 19.35 | BA.1.1 | 0.36 | 136X | 1380 |
| 322 | hCoV-19/Kazakhstan/NLA/barcode51-18-MN908947.3/2022 | EPI_ISL_13717992 | 2022-01-15 | 30 | Female | 18.86 | BA.1.1 | 0.36 | 142X | 1484 |
| 323 | hCoV-19/Kazakhstan/NLA/barcode52-18-MN908947.3/2022 | EPI_ISL_13717993 | 2022-01-15 | 53 | Female | 18 | BA.1.1 | 0.36 | 144X | 1345 |
| 324 | hCoV-19/Kazakhstan/NLA/barcode53-18-MN908947.3/2022 | EPI_ISL_13717994 | 2022-01-15 | 32 | Male | 15.93 | BA.1.1 | 0.36 | 182X | 1396 |
| 325 | hCoV-19/Kazakhstan/NLA/barcode54-18-MN908947.3/2022 | EPI_ISL_13717995 | 2022-01-17 | 34 | Male | 13.98 | BA.1.1 | 0.36 | 220X | 1332 |
| 326 | hCoV-19/Kazakhstan/NLA/barcode55-18-MN908947.3/2022 | EPI_ISL_13717996 | 2022-01-17 | 11 | Male | 17.31 | BA.1.1 | 0.36 | 241X | 1317 |
| 327 | hCoV-19/Kazakhstan/NLA/barcode67-18-MN908947.3/2022 | EPI_ISL_13717997 | 2022-01-13 | 66 | Female | 25.01 | BA.1.1 | 0.36 | 204X | 1304 |
| 328 | hCoV-19/Kazakhstan/NLA/barcode68-18-MN908947.3/2022 | EPI_ISL_13717998 | 2022-01-19 | 26 | Male | 21.65 | BA.1.1 | 0.35 | 112X | 2469 |
| 329 | hCoV-19/Kazakhstan/NLA/barcode69-18-MN908947.3/2022 | EPI_ISL_13717999 | 2022-01-19 | 40 | Male | 23.7 | BA.1.1 | 0.35 | 133X | 2193 |
| 330 | hCoV-19/Kazakhstan/NLA/barcode70-18-MN908947.3/2022 | EPI_ISL_13718000 | 2022-01-19 | 53 | Female | 20.26 | BA.1.1 | 0.36 | 143X | 1612 |
| 331 | hCoV-19/Kazakhstan/NLA/barcode75-18-MN908947.3/2022 | EPI_ISL_13718001 | 2021-09-02 | unknown | unknown | unknown | AY.122 | 0.36 | 96X | 1866 |
| 332 | hCoV-19/Kazakhstan/NLA/barcode80-18-MN908947.3/2022 | EPI_ISL_13718002 | 2022-01-19 | 34 | Female | 19.69 | BA.1.1 | 0.36 | 152X | 1480 |
| 333 | hCoV-19/Kazakhstan/NLA/barcode81-18-MN908947.3/2022 | EPI_ISL_13718003 | 2022-01-20 | 54 | Female | 21.62 | BA.1.1 | 0.35 | 74X | 2605 |
| 334 | hCoV-19/Kazakhstan/NLA/barcode82-18-MN908947.3/2022 | EPI_ISL_13718004 | 2022-01-20 | 33 | Male | 22.51 | BA.1.1 | 0.36 | 102X | 1650 |
| 335 | hCoV-19/Kazakhstan/NLA/barcode83-18-MN908947.3/2022 | EPI_ISL_13718005 | 2022-01-20 | 28 | Female | 21.66 | BA.1.1 | 0.36 | 118X | 1444 |
| 336 | hCoV-19/Kazakhstan/NLA/barcode84-18-MN908947.3/2022 | EPI_ISL_13718006 | 2022-01-20 | 50 | Male | 21.1 | BA.1 | 0.34 | 86X | 2815 |
| 337 | hCoV-19/Kazakhstan/NLA/barcode85-18-MN908947.3/2022 | EPI_ISL_13718007 | 2021-09-02 | unknown | unknown | unknown | B.1.1.7 | 0.37 | 94X | 936 |
| 338 | hCoV-19/Kazakhstan/NLA/barcode87-18-MN908947.3/2022 | EPI_ISL_13718008 | 2021-09-02 | unknown | unknown | unknown | AY.122 | 0.37 | 279X | 384 |
| 339 | hCoV-19/Kazakhstan/NLA/barcode91-18-MN908947.3/2022 | EPI_ISL_13718009 | 2022-01-21 | 37 | Male | 26.33 | BA.1.1 | 0.36 | 186X | 1386 |
| 340 | hCoV-19/Kazakhstan/NLA/barcode93-18-MN908947.3/2022 | EPI_ISL_13718010 | 2022-01-10 | 61 | Male | 28.5 | BA.1.1 | 0.36 | 212X | 1282 |
| 341 | hCoV-19/Kazakhstan/NLA/barcode94-18-MN908947.3/2022 | EPI_ISL_13718011 | 2022-01-20 | 21 | Female | 22.9 | BA.1.1 | 0.36 | 191X | 1332 |
